# Beneficial effects of food-medicine homologous herbs for patients with diabetic kidney disease

**DOI:** 10.3389/fimmu.2025.1698240

**Published:** 2025-12-11

**Authors:** Qin Zeng, Wenru Wang, Yihan Li, Ming Wang, Zhengtang Liu, Lei Yan, Jingyi Zhan, Xinhui Wang, Ying Liang, Renhuan Yu

**Affiliations:** 1 Department of Nephrology, Xiyuan Hospital of China Academy of Chinese Medical Sciences, Beijing, China; 2 Zhujiang Hospital, Southern Medical University, Guangzhou, China; 3 Department of Geriatrics, Xiyuan Hospital of China Academy of Chinese Medical Sciences, Beijing, China

**Keywords:** medicine and food homology, food-medicine homologous herbs, diabetic kidney disease, bioactive components, dietary supplements

## Abstract

**Objectives:**

Global diabetes rates are rising sharply, driving a parallel increase in diabetic kidney disease (DKD) - a key diabetic complication. This trend poses mounting public health and economic burdens worldwide. Current therapies remain inadequate, making DKD progression a pressing unmet need. This review aims to assess the efficacy and molecular mechanisms of food-medicine homologous herbs for DKD treatment.

**Methods:**

A comprehensive literature search was conducted across multiple databases (PubMed, Web of Science, Cochrane Library, and Embase) from inception to March 2025, using keywords including “diabetic kidney disease”, and “traditional Chinese medicine”. The search was meticulously designed to cover relevant research extensively. Data extraction focused on herb names, bioactive compounds, experimental models, therapeutic effects, and molecular targets.

**Findings:**

This review highlights 29 food-medicine homologous herbs with proven safety and efficacy in DKD. These herbs alleviate immune-inflammatory responses by modulating NF-κB, interleukins, TNF-α, chemokines, and adhesion molecules. They also reduce mitochondrial and non-mitochondrial ROS production, improving oxidative stress via Keap1/Nrf2/ARE, AMPK/SIRT, and NF-κB pathways. Renal fibrosis is suppressed through targeting fibrosis markers and regulating TGF-β/Smad and Notch signaling. Additionally, these herbs inhibit the AGEs/RAGE axis, correct gut dysbiosis, reduce apoptosis, activate autophagy, inhibit ferroptosis, and modulate microRNAs, collectively exerting renoprotective effects in DKD.

**Conclusions:**

Food-medicine homologous herbs demonstrate properties that align well with medical nutrition therapy principles, offering novel adjunctive therapeutic options for DKD.

## 1 Introduction

Diabetic kidney disease (DKD), is one of the most significant microvascular complications of diabetes. It affects approximately 30% to 40% of diabetic patients and remains the leading cause of chronic kidney disease (CKD) globally (1). By 2045, an estimated 693 million people worldwide will have diabetes (2). The rising prevalence of DKD closely parallels the dramatic increase in diabetes, making DKD a major yet under-recognized contributor to the global burden of disease.

The pathogenesis of diabetic nephropathy is the combined result of a series of intertwined abnormalities in metabolic, inflammatory, oxidative stress, and cellular signaling pathways (3). Persistent hyperglycemia activates metabolic pathways such as the polyol pathway and advanced glycation end products, leading to mitochondrial dysfunction and excessive reactive oxygen species (ROS) production. This, in turn, damages the glomerular basement membrane and podocyte structures, resulting in increased permeability of the filtration barrier (4). Concurrently, ROS promotes the activation of transcription factors such as nuclear factor κB (NF-κB), inducing the secretion of inflammatory mediators like tumor necrosis factor-alpha (TNF-α) and interleukins (ILs). This attracts the infiltration of immune cells including macrophages and T cells, establishing a chronic inflammatory microenvironment that accelerates extracellular matrix deposition and fibrosis (5). The renin-angiotensin system further amplifies hemodynamic stress during this process, promoting glomerular hyperfiltration and increased capillary wall tension. This activates the Notch, transforming growth factor beta 1/Smad (TGF-β1/Smad), and endothelin-1 signaling pathways, leading to mesangial cell proliferation and matrix accumulation (6). Dysregulated lipid metabolism also plays a pivotal role. Abnormal fatty acid accumulation activates SREBP-1, inhibits PPAR-α/FXR, generates lipotoxicity, and exacerbates oxidative stress and inflammatory responses. Concurrently, it disrupts autophagy processes, preventing the efficient clearance of damaged organelles and further worsening tubulointerstitial injury (7).The core pathogenesis of DKD involves metabolic disturbances and hemodynamic abnormalities induced by chronic hyperglycemia, which subsequently activate multiple pathways including oxidative stress, inflammation, and pro-fibrotic signaling, ultimately leading to glomerulosclerosis and tubulointerstitial fibrosis.

Guidelines from the Kidney Disease: Improving Global Outcomes (KDIGO) and the American Diabetes Association recommend a range of treatments, including renin-angiotensin system inhibitors, statins, metformin, sodium-glucose cotransporter 2 inhibitors (such as empagliflozin), glucagon-like peptide-1 agonists (such as liraglutide), and nonsteroidal mineralocorticoid receptor antagonists (such as finerenone) (8). However, despite these therapeutic options, the progression of DKD remains a significant risk (9). For instance, long-term statin use may increase insulin resistance, disrupt lipid metabolism, induce inflammation, and fibrosis, potentially accelerating DKD progression in diabetic mice (10). Consequently, there is an urgent need for innovations in therapeutic drug development and biomarker discovery to improve patient outcomes. Several treatments, such as endothelin receptor antagonists and dipeptidyl peptidase-4 inhibitors, are currently in clinical trials and awaiting further efficacy and safety evaluations (11, 12). Additionally, early-stage cell therapies, such as renal autologous cells and mesenchymal stem cells, are under investigation (13). As of June 5, 2025, 663 DKD-related clinical trials have been registered on ClinicalTrials.gov. These trials explore a wide range of interventions, including oral and sedative infusions of western medicines, cellular injections, acupuncture, herbal remedies, and care optimization strategies. This broad global effort underscores the importance of addressing DKD as a major public health issue.

In addition to pharmacotherapy, lifestyle modifications play a critical role in managing DKD. These include regular exercise (KDIGO recommends 150 minutes of moderate to high-intensity activity per week) and the cessation of smoking and alcohol consumption. Dietary modifications are also crucial, and KDIGO emphasizes the importance of medical nutrition therapy. Some studies recommend a ketogenic diet for DKD patients, characterized by high fat, moderate protein, and very low carbohydrate intake (less than 50 g/day) (14). Another widely recommended dietary approach for DKD is the Mediterranean diet, which includes vegetables, fruits, nuts, legumes, whole grains, and olive oil, along with moderate consumption of fish and poultry, and low intake of sweets, red meat, and dairy products (15). These diets are thought to reduce renal inflammation and oxidative stress in DKD patients, potentially improving renal function (16). However, some high-quality studies have indicated that the Mediterranean diet may not prevent DKD, possibly due to low adherence (17, 18). Additionally, the Indo-Mediterranean diet, which incorporates whole grains, millet, porridges, legumes, and spices, has been shown to enhance the antioxidant properties of the Mediterranean diet (19). A key area that may enhance dietary management for DKD is recognizing the therapeutic and medicinal properties of food. This could significantly improve the effectiveness of medical nutritional therapy for type 2 diabetes.

**Figure 1:**
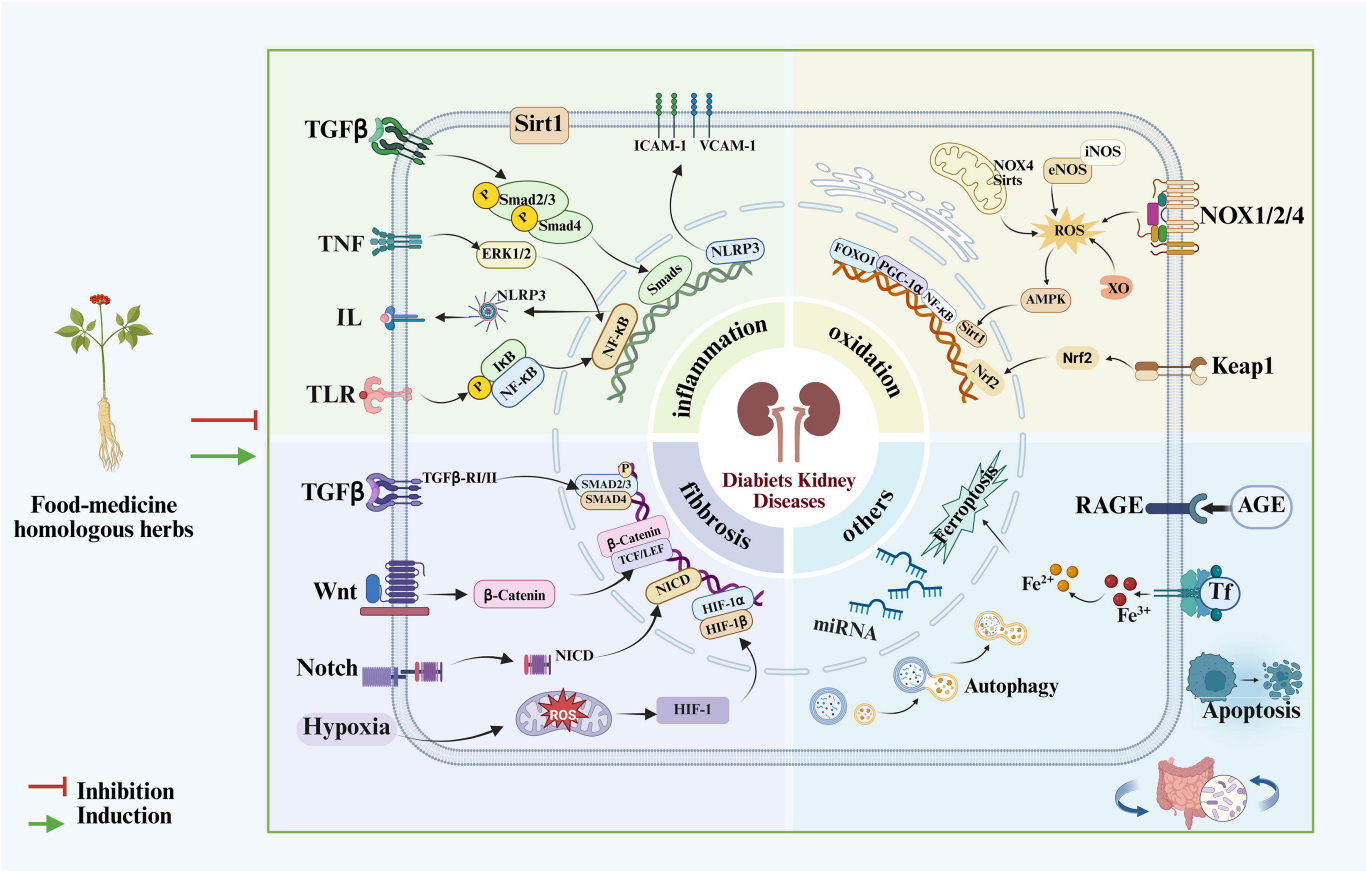
Mechanism diagram of food-medicine homologous herbs in DKD.

In the West, there’s the old adage, “*medicine and food homology*” (a misquote often attributed to Hippocrates of Kos). In the 21st century, the United States has a strong interest in food as medicine interventions in health care practice and policy (20). In China, a similar concept exists called “medicine and food homology”, which posits that many substances share a common origin for both dietary and medicinal purposes (21). Increasing evidence supports the therapeutic effects of substances that serve as both food and Chinese medicine, which are recommended as functional foods or dietary supplements for DKD patients (22, 23). Functional foods or dietary supplements can provide DKD patients with additional nutrients or bioactive compounds to support their health management, and these food-medicine homologous herbs represent a valuable resource for creating them, warranting further summarization and exploration. One case-control study found that a higher dietary variety may be associated with a lower incidence of DKD (24). Accordingly, this review centers on food-medicine homologous herbs listed in the “*Management Catalogue of Substances that are both Food and Chinese Herbal Medicine*”, issued by the National Health Commission of China. A comprehensive literature search was conducted across PubMed, Web of Science, Cochrane Library, and Embase to identify English-language studies on food-medicine homologous herbs for the treatment of DKD, covering all available publications through March 2025. Search strategies incorporated both Medical Subject Headings and free-text terms to ensure a robust retrieval of relevant literature. Relevant articles were subsequently screened and evaluated to extract additional sources through manual reference checking. Both basic experimental studies and clinical investigations, including randomized controlled trials, were included. This paper aims to summarize the efficacy and potential targets of food-medicine homologous herbs in treating DKD, highlighting their bioactive components (**Table 1**, **Figure 1**). Ultimately, this could promote the development of medical nutrition therapies, enrich dietary options for DKD patients, improve dietary adherence, and provide insights for developing therapeutic drugs for DKD.

**Table 1:** The bioactivities and potential targets of food-medicine homologous herbs and their main active ingredients in the treatment of diabetic kidney disease.

Herbs (Pinyin)	Scientific name	Family	Consumed part	Main active ingredients	Models	Bioactivities	Targets
Chinese Yam (Shan Yao) (25–27)	*Dioscorea opposita* Thunb.	*Dioscoreaceae*	Rhizome	Dioscin; Dioscorea zingiberensis; Dioscorea villosa;	FRU-induced renal damage in rats; HFD and STZ-induced DN mice;	Anti-inflammatory	↑Sirt3 ↓IL-1β, NLRP3, Caspase-1, IL-6, TNF-α, HMGB-1, COX-2, c-Jun, c-Fos, NF-κB
						Anti-oxidative	↑SOD2, CAT, GST, nuclea Nrf2, Sirt3 ↓Keap1, 8-OHdG, MDA, p66Shc
						Anti-fibrosis	↑Smad7 ↓α-SMA, COL1A1/3A1, TGF-β1, p-Smad3 ↑α-SMA, vimentin
Hawthorn (Shan Zha) (28, 29)	*Crataegus pinnatifida* Bunge	Rosaceae	Sarcocarp	Hyperoside	HG-induced podocyte and HMCs; STZ-induced DN rats	Anti-inflammatory	↓IL-1β, TNF-a, MCP-1
						Inhibit cell proliferation	↓ERK/CREB/miR-34a
						Attenuate apoptosis	↑Bcl-2, Caspase-3 ↓Bax
						Increase miRNA	↑miR-499e-5p ↓APC
Licorice (Gan Cao) (30–39)	*Glycyrrhiza alalensis* X.Y. Li	Fabaceae	Rhizome	18α-Glycyrrhetinic acid; Isoliquiritigenin; Isoangustone A Licochalcone A; Glabridin; Licorice extracts;	FRU-incubated HK2 cells; FRU-induced mice; STZ-induced DN rats; AGE challenge in HK-2 cells; HG-induced HMCs; STZ-induced DN mice; HFD and STZ-induced DN rats; HG-induced NRK-52E cells;	Anti-inflammatory	↑IL-10 ↓IL-6, IL-18, IL-1β, TNF-α, CCL-3, ICAM-1, MCP-1, F4/80, CD68, PGE2, p-IKκα, p-IκBα, p-NF-κB (p65), NF-κB NLRP3
						Anti-oxidative	↑TAC, SOD, SOD1, CAT, HO-1, NQO-1, GCLM, nuclear Nrf-2, GSH, GSH-Px, Sirt1, ATP ↓ROS, H^2^O^2^, O^2^, 8-OHdG, MDA, iNOS, XO, cytoplasm Nrf-2, p-p38, ERK1/2, JNK
						Anti-fibrosis	↑E-cadherin, MMP-2, MT-1MMP, Smad7, Delta-1/Jagged-1, NICD2, MAML-1 ↓ Fibronectin, α-SMA, vimentin, col IV, COL1A, CTGF, TIMP-1/2, p-STAT3, STAT3, Smad4, p-Smad2, TGF-β1, TGF-βRI/RII, PKCβ2, HIF-1α
						Inhibit ferroptosis	↑GPX4, SLC7A11, SLC3A2 ↓Kidney iron content, TFR1, VEGF, Akt, p-Akt, ERK1/2, p-ERK1/2
						Inhibite AGEs/RAGE axis	↓AGEs/RAGE
Semen Cassiae (Jue Ming Zi) (40)	*Cassia obtusifolia* L.	Fabaceae	Seed	Cassiae Semen extract	STZ-induced DN rats	Anti-inflammatory	↓IL-1β, IL-6, TNF-α
						Anti-oxidative	↑SOD, CAT, GSH-Px ↓MDA
						Inhabit RAGE	↓RAGE
Semen Phaseoli Angularis (Chi Xiao Dou) (41, 42)	*Vigna umbellata* (Thunb.) Ohwi & H. Ohashi	Fabaceae	Seed	Natto-red beans extract; Azuki bean seed coats	STZ-induced DN rat	Anti-inflammatory	↓CRP, IL-6, TNF-α, MCP-1
						Anti-oxidative	↑GSH, SOD ↓ROS, GPx, MDA
						Anti-fibrosis	↓ MCP-1
						Inhabit AGEs	↓AGEs
Monk fruit (Luo Han Guo) (43–45)	*Siraitia grosvenorii* (Swingle) C. Jeffrey ex A.M. Lu & Zhi Y. Zhang	Cucurbitaceae	Fruit	Siraitia grosvenorii polysaccharide; Mogroside IIIE; Momordica grosvenori extract;	CdCl2-induced DN mice; HG-induced MPC-5; DN mice (Intra-peritoneal injection of alloxan)	Anti-inflammatory	↓ IL-1β, IL-6, TNF-α, TLR4, NF-κB (p65), NLRP3, ASC, Caspase-1
						Anti-oxidative	↑SOD, CAT, AMPK / Sirt1, GSH, Mn-SOD, GSH-Px, HO-1 ↓MDA
						Attenuate apoptosis	↑Bcl-2, AMPK / Sirt1 ↓Bax, Caspase-3, Caspase-9
Flos Lonicerae (Jin Yin Hua) (46–50)	*Lonicera japonica* Thunb.	Caprifoliaceae	Stem, leaf, flower	Ethanol Extract of Lonicera japonica; Luteolin; The polysaccharides from LJP;	Db/db mice; HG-induced MPC-5; STZ-induced DN rats	Anti-inflammatory	↑IL-10 ↓IL-1β, IL-6, IL-17A, TNF-α, NLRP3, Caspase-1, TGF-β1
						Anti-oxidative	↑ CAT, SOD, GSH, HO-1 ↓ROS, MDA, STAT3, AKT/PKB
						Anti-fibrosis	↓Fn, α-SMA, col I/IV, STAT3, cyclin D1, c-myc
						Attenuate apoptosis	↑Bcl-2 ↓Caspase-3, Caspase-9, Caspase-6
Ginger (Jiang) (51–58)	*Zingiber officinale* Roscoe	Zingiberaceae	Rhizome, cork	Zingerone; Zingiber officinale; 6-Gingerol; 6-Shogaol; Zerumbone; Hesperetin;	Db/db mice; HFD and STZ-induced DN rats; Glucose-incubated HK-2 cells; STZ-induced DN rats; HG-Induced Immortalized human podocytes;	Anti-inflammatory	↓ CRP, IL-1β, IL-6, TNF-α, TGF-β, NF-κB, NF-κB (p65), MCP-1, ICAM-1
						Anti-oxidative	↑CAT, SOD, GST, GSH, GPx, GR, nuclear Nrf2 ↓ROS, MDA, NOX4, GSSG, Protein carbonyl
						Anti-fibrosis	↓Fn, vimentin, α-SMA, col IV, TGF-β1
						Attenuate apoptosis	↓Cytochrome c, Caspase-3
Fructus Lycii (Gou Qi) (59–64)	*Lycium barbarum* L.	Solanaceae	Fruit	Lycium barbarum polysaccharides; Zeaxanthin	HFD and STZ-induced DN mice; ALX-induced DN rabbits; HFHSD and STZ-induced DN rats	Anti-inflammatory	↓SAA3, IL-1β, IL - 2, IL-6, AngII, TNF-α, IFN -α, MCP-1, ICAM-1, IκBα, NF-κB
						Anti-oxidative	↑SOD, GSH-Px, CAT, GST, GSH, GPx, GSH-Px, GR ↓MDA, IκBα, NF-κB, ERK1/2, PKC
Fructus Gardeniae (Zhi Zi) (65)	*Gardenia jasminoides* J. Ellis	Rubiaceae	Fruit	Genipin-1-β-d-gentiobioside	HFD and STZ-induced DN mice; HG-induced podocyte model	Anti-inflammatory	↓IL-1β, IL-6, TNF-α, NLRP3, ASC, Caspase-1, GSDMD-N, p-NF-κB
						Anti-oxidative	↓MDA
Fructus Mori (Sang Shen) (66, 67)	*Morus alba* L.	Moraceae	Fruit	Mulberry extract; Black mulberry fruit extract	Diabetic nephropathy patients; STZ-induced DN rats	Anti-inflammatory	↓HSCRP, TNF-α, VCAM-1
						Anti-oxidative	↑GSH, CAT ↓MDA
						Anti-fibrosis	↓Fn
Folium Mori (Sang Ye) (68–84)	*Morus alba* L.	Moraceae	Leaf	Mulberry leaves extracts; Morus alba L. (Sangzhi) alkaloid; Sang Tong Jian; Mulberry leaf polysaccharides; Mulberry leaf, polysaccharides and alkaloids; The combination (MAF1:1 and MAF1:5) of mulberry leaf alkaloids and flavonoids extract; Mulberry leaves extracts;	High-fat mice; HG-incubated HK2 cells; Zucker diabetic fatty (ZDF) rats; HFD and STZ-induced DN mice/rats; STZ-induced DN rats; Db/db mice; HG-Induced Immortalized human podocytes;	Anti-inflammatory	↓ CRP, IL-1β, IL-6, TNF-α, TRAF6, MCP - 1, NF-κb, NF-κB (p65)
						Anti-oxidative	↑GSH, G6PDH, GR, GST, SOD, GSH-Px, Serum FRAP ↓MDA, serum/ kidney TBARS
						Anti-fibrosis	↑E-cadherin, Smad7 ↓Fibronectin, α-SMA, col IV, col IV, connexin 43, CTGF, TGF-β1, Smad2/3/4, p-Smad2/3
						Regulate gut dysbiosis	↑↓Gut microbiota, serum and urine metabolome ↑Fecal bile acids, SCFAs
						Inhabit nitrosylation	↓The nitrate/nitrite content
Radix Platycodonis (Jie Geng) (85)	*Platycodon grandiflorus* (Jacq.) A. DC.	Campanulaceae	Root	Platycodin D	HFD and STZ-induced DN mice; HG-induced RAW264.7 cells; HG-induced HK2 cells	Anti-inflammatory	↓IL-1β, IL-6, TNF-α, IκBα, IKKβ, NF-κB,
						Anti-oxidative	↓ROS
						Attenuate apoptosis	↑Bcl-2, Bcl-XL ↓Bax, Caspase-3, Caspase-9, PI3K/AKT
Radix Puerariae (Ge Gen) (86–94)	*Pueraria lobata* (Willd.) Ohwi	Fabaceae	Root	Puerarin	STZ-induced DN mice/rats; STZ-induced DN in the endothelial nitric oxide synthase-null (eNOS) mice; HG-induced podocyte model; HG-incubated HK2 cells	Anti-inflammatory	↑IL-4, Sirt1 ↓IL-6, TNF-α, IFN-γ, ICAM-1, NF-κB
						Anti-oxidative	↑SOD, Mn-SOD, CAT, GSH-Px, Sirt1/FOXO1, PGC-1α ↓ROS, 8-OHdG, MDA, NO, NOX4, NF-κB, eNOS
						Anti-fibrosis	↓Fn, col IV, CTGF, TGF-β1, Smad2, MMP-9, c-fos/c-jun
						Activate autophagy	↑LC3, Beclin-1, Atg5, AMPK/Sirt1, PERK/eIF2α/ ATF4 ↓p62, LKB1 acetylation
Herba Taraxaci (Pu Gong Ying) (95)	*Taraxacum mongolicum* Hand. - Mazz.	Asteraceae	whole herb	Dandelion sterol	STZ-induced DN rats; HG-induced HK2 cells	Anti-inflammatory	↓IL-1β, IL-6, TNF-α, NF-κB (p65)
Fructus Rubi (Fu Pen Zi) (96, 97)	*Rubus chingii* Hu	Rosaceae	Fruit	Raspberry Extracts; Pelargonidin-3- O-glucoside	Db/db mice	Anti-inflammatory	↓IL-1β
						Regulate gut dysbiosis	↑↓Gut microbiota ↑Fecal SCFAs
Radix Ginseng (Ren Shen) (98–101)	*Panax ginseng* C.A. Mey.	Araliaceae	Root	Ginsenoside Rg1; Ginsenoside Rh1; 20(R)-ginsenoside Rg3	HG-induced HBZY-1 cells; STZ-induced DN rats; HFD and STZ-induced DN mice	Anti-inflammatory	↓TNF-α, IL-1β, IL-6, p-IκB α, p- IKκβ, p-NF-κB
						Anti-oxidative	↑SOD, CAT, HO-1, GSH, p-AMPKα ↓MDA, MAPKs, NOX1/4, PI3K/AKT
						Anti-fibrosis	↓Fn, CN, TGF-β, p-Smad2/3, NFAT2, TRPC2, p-PLC, CD36
						Attenuate apoptosis	↑Bcl-2, Bcl-XL, p-AMPKα ↓Bax, cytochrome c, Caspase-3, Caspase-8, Caspase-9, PI3K/AKT
						Inhabit AGEs	↓AGEs
Spica Prunellae (Xia Ku Cao) (102)	*Prunella vulgaris* L.	Lamiaceae	Fruit	Prunella vulgaris	HG-induced HMCs; STZ-induced diabetic rats	Anti-inflammatory	↓ICAM-1, MCP-1, p-IκBα, NF-κB (p65)
						Anti-oxidative	↓ROS
						Anti-fibrosis	↑Smad7 ↓col IV, CTGF, TGF-β1, Smad-2/4
Rhizoma Curcumae Longae (Jiang Huang) (103, 104)	*Curcuma Longa* L.	Zingiberaceae	Rhizome	Turmeric; Curcumin	Type 2 diabetic nephropath patients; HG-induced GMCs	Anti-inflammatory	↓IL-8
						Anti-fibrosis	↓Fn, TGF-β1, SphK1, S1P
Cortex Cinnamomi (Rou Gui) (105)	*Cinnamomum cassia* (L.) J. Presl	Lauraceae	bark	The water extract of Chinese cinnamon	HG-induced MCs	Anti-fibrosis	↓Fn, col IV
Semen Euryales (Qian Shi) (106, 107)	*Euryale ferox* Salisb. ex K.D. Koenig & Sims	Nymphaeaceae	Seed	Ungerminated seed extract; germinated seed extract; Compounds of E. ferox seeds	STZ-induced DN mice; HG-induced MCs	Anti-oxidative	↑TAC, SOD, CAT, HO-1, nuclear Nrf2, mTOR ↓ ROS, MDA, GSH, Keap1, AMPK
Folium Nelumbinis (He Ye) (108)	*Nelumbo nucifera* Gaertn.	Nelumbonaceae	Leaf	Nelumbo nucifera leaf extract	HFD and STZ-induced DN rats	Anti-oxidative	↑SOD, CAT, GSH, GPx, nuclear Nrf2 ↓ROS
Rhizoma Polygonati (Huang Jing) (109)	*Polygonatum cyrtonema* Hua	Asparagaceae	Rhizome	Syringaresinol-di-O-β-D-glucoside	STZ-induced DN mice	Anti-oxidative	↑TAC ↓MDA
Folium Perillae (Zi Su) (110)	*Perilla frutescens* (L.) Britton	Lamiaceae	Leaf or fresh shoots	Perilla frutescens sprout extract	HG-induced MMCs	Anti-oxidative	↑SOD, GSH ↓ROS, AMPK, NOX2/4
						Anti-fibrosis	↓Fibronectin, col I/IV, NOX2/4, AMPK
Semen Sesami Nigrum (Hei Zhi Ma) (111)	*Sesamum indicum* L.	Pedaliaceae	Seed	Sesame oil	STZ-induced DN rats	Anti-oxidative	↑GSH, Vitamin C/E
Herba Menthae (Bo He) (112)	*Mentha haplocalyx* Briq.	Lamiaceae	Aboveground parts of herbs	Methanolic Mentha longifolia	Serotonin−Induced in diabetic rats	Anti-oxidative	↑SOD, CAT ↓ROS
Radix Angelicae Sinensis (Dang Gui) (113)	*Angelica sinensis* (Oliv.) Diels	Apiaceae	Root	The concentrated granules of DangGui	STZ-induced DN rats; Glucose-incubated rat MCs	Anti-oxidative	↓ROS
Radix Chaenomeles (Mu Gua) (114)	*Chaenomeles sinensis* (Thouin) Koehne	Rosaceae Juss	Fruit	Chinese quince extract	Diabetic KK-A(y) mice	Inhabit AGEs	↓AGEs
Semen Alpiniae Oxyphyllae (Yi Zhi Ren) (115)	*Alpinia oxyphylla* Miq.	Zingiberaceae	Fruit	Alpinia oxyphylla Miq. extract	Db/db mice	Target miRNAs	↑Let-7k, miR-378d, miR-129-1-3p, miR-21a-5p, miR-29c-3p, miR-203-3p, miR-7a-5p

↑, upregulate; ↓, downregulate.

## 2 Theoretical foundation and developmental context of the concept of medicine and food homology

The concept of medicine and food homology represents one of the most distinctive theoretical frameworks in TCM. This philosophy can be traced back more than two millennia to ancient classical texts like *Huangdi Neijing* (The Yellow Emperor’s Inner Canon), where the principles of “preventive treatment of disease” (zhi wei bing) and “using food as medicine” were emphasized as fundamental strategies for health preservation (116, 117). Within this framework, dietary regulation is regarded as the primary approach to maintaining physiological harmony and preventing illness. Subsequent classical works, including the *Shennong Bencao Jing* (The Divine Farmer’s Classic of Materia Medica) and the *Nan Jing* (The Classic of Difficult Issues), further elaborated on this foundation (118). These texts systematically documented grains, vegetables, fruits, and medicinal herbs, highlighting that foods not only provide essential nutrients but also possess pharmacological properties similar to medicinal substances. Collectively, these early records established the dual tenet that “food can serve as medicine, and medicine can also serve as food” (119, 120).

This doctrine continued to evolve across successive dynasties. During the Tang Dynasty, the eminent physician Sun Simiao devoted an entire chapter to “dietary therapy” in his seminal work *Qianjin Yaofang* (Essential Prescriptions Worth a Thousand Gold for Emergencies), advocating the use of food as the first line of treatment before resorting to pharmaceuticals (121). By the Ming Dynasty, Li Shizhen’s *Bencao Gangmu* (Compendium of Materia Medica) further advanced this concept, establishing a systematic classification of foods and herbs with dual medicinal and nutritional functions. His work not only consolidated prior knowledge but also greatly expanded the pharmacognostic understanding of substances that bridge the boundary between diet and medicine (122).

The concept of medicine and food homology posits that numerous natural substances can serve simultaneously as food and medicine. Its core philosophy lies in recognizing the intrinsic connection between diet and pharmacotherapy, emphasizing a holistic approach to health maintenance and disease prevention through balanced nutrition. In the theoretical framework of traditional Chinese medicine (TCM), both foods and medicinal substances possess inherent properties and flavors, namely the four natures (cold, hot, warm, cool) and the five flavors (sour, bitter, sweet, pungent, salty) (123). Importantly, the five flavors are believed to correspond to specific visceral organs and exert targeted physiological functions: the sour flavor corresponds to the liver, the bitter to the heart, the sweet to the spleen, the pungent to the lungs, and the salty to the kidneys. This classical doctrine of the “five flavors entering the five organs” forms the theoretical foundation for selecting food-medicine homologous herbs in interventions for DKD.

According to TCM theory, the pathogenesis of DKD primarily stems from spleen-kidney deficiency, which leads to impaired fluid metabolism and accumulation of dampness and turbidity. Consequently, herbs with a sweet flavor are used to tonify the spleen and replenish qi, while those with a salty flavor are believed to direct therapeutic effects toward the kidneys. Recent pharmacological studies have demonstrated that several commonly used food-medicine homologous herbs, including *Dioscorea opposita* Thunb. (Dioscoreaceae), *Zingiber officinale* Roscoe (Zingiberaceae), *Perilla frutescens* (L.) Britton (Lamiaceae), and *Panax ginseng* C.A. Mey. (Araliaceae), can effectively alleviate renal inflammation and oxidative stress in DKD (21, 46, 93, 105).

In contemporary practice, the application of food-medicine homologous herbs has been standardized and regulated. In 2020, the National Health Commission of China released the most recent official list, comprising 102 approved food-medicine homologous herbs (119). This list clearly specifies the botanical sources, pharmacological functions, and recommended safe dosage ranges of these materials, thereby establishing a robust and standardized framework for their safe application in the management of chronic diseases such as DKD. This regulatory foundation also underpins the discussion in the present review regarding the therapeutic potential of food-medicine homologous herbs in DKD management.

## 3 Food-medicine homologous herbs with therapeutic effects on DKD

### 3.1 Anti-inflammation

Traditionally considered non-inflammatory, DKD has revealed complex inflammatory signaling pathways through comprehensive genome and transcriptome sequencing analyses (124). Inflammation is now recognized as a central feature in the pathogenesis of DKD and plays a critical role in the progression of kidney injury (125). Increasing clinical and experimental evidence links DKD to the accumulation of inflammatory cells, particularly T cells and macrophages, which infiltrate the glomerulus and interstitium, leading to renal tissue damage (126). When renal cells are damaged, they release endogenous danger signals, activating innate immunity and triggering a cascade of inflammatory responses. As DKD advances, impaired immune function further heightens patient susceptibility to infections. Inflammatory cells, along with their associated products such as transcription factors, cytokines, chemokines, and adhesion molecules, further exacerbate the altered renal microenvironment.

**Figure 2:**
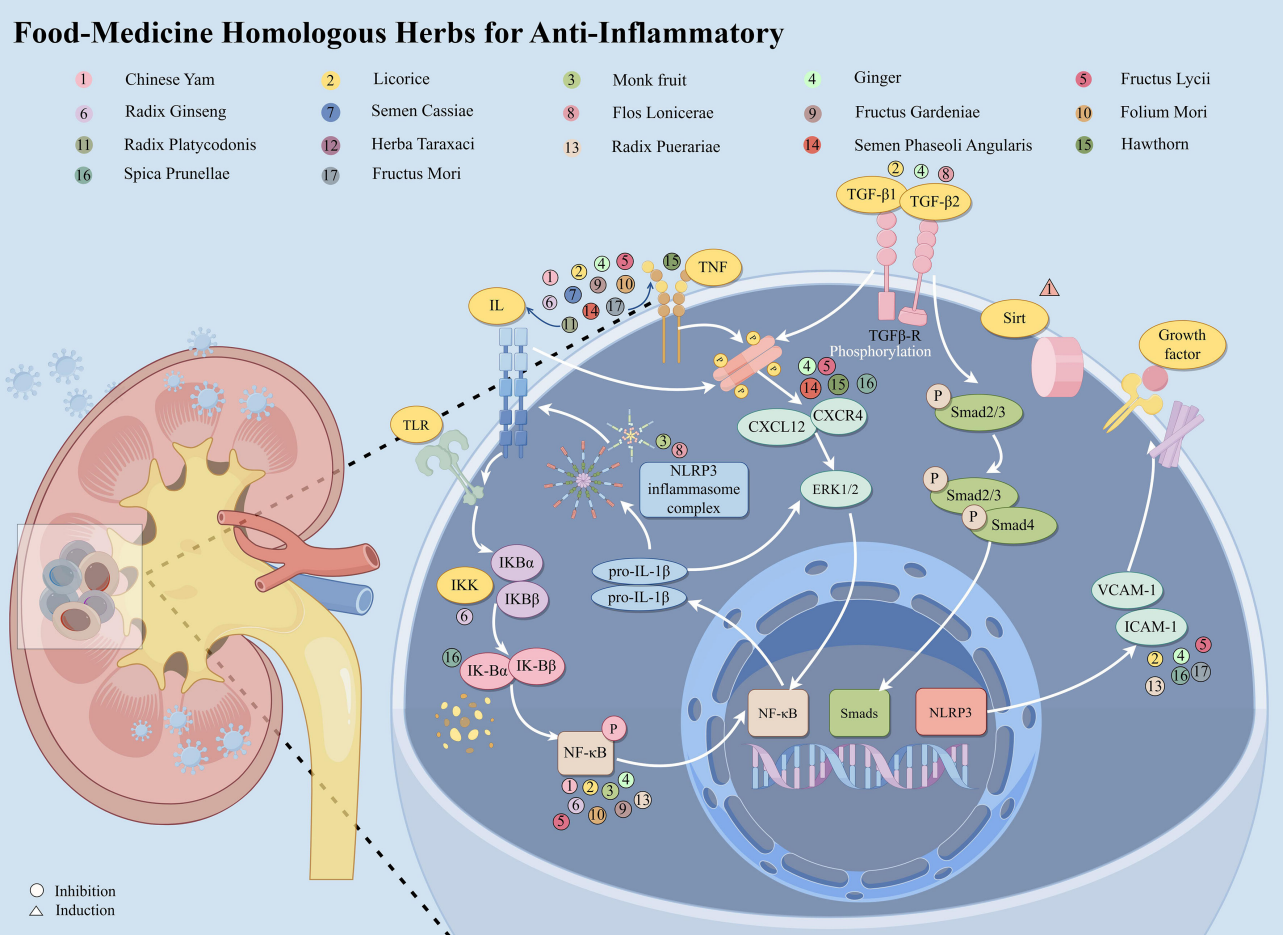
Food-medicine homologous herbs protect against DKD by alleviating inflammation.

Given these factors, there is an urgent need for therapeutic strategies capable of modulating immune-inflammatory responses with improved safety profiles. Currently, interventions specifically targeting inflammatory mediators to slow DKD progression are limited. However, there is substantial evidence supporting the therapeutic potential of several herbal medicines with anti-inflammatory properties. These herbs can inhibit inflammation through various mechanisms targeting specific pathways (127–129). For example, *Panax ginseng* C.A. Mey. (Araliaceae) has shown significant potential in reducing DKD-related inflammation (130). Focusing on anti-inflammatory herbs derived from natural medicinal and dietary sources presents a promising approach to address inflammation in DKD. These herbs have the potential to target specific inflammatory mediators and intracellular signaling pathways, offering viable treatment strategies. This section explores the impact of medicinal herbs on key inflammatory pathways in DKD, such as NF-κB, ILs, TNF-α, chemokines, adhesion molecules, and TGF-β1/Smad signaling pathways (**Figure 2**).

#### 3.1.1 Regulating NF-κB signaling pathway

NF-κB is a critical transcription factor involved in regulating processes such as cell proliferation, differentiation, inflammation, apoptosis, and immune responses. Normally, NF-κB remains inactive due to its association with the inhibitor of NF-κB (IκB). Upon cellular stimulation by various triggers—including pathogenic microorganisms, stress, or inflammatory mediators—NF-κB is activated through the inhibitor of κB kinase (IKK) complex. This process is mediated by NF-κB-inducing kinase (NIK), which phosphorylates IKKα and IKKβ, ultimately leading to the activation of NF-κB. Once activated, NF-κB promotes the expression of inflammatory factors such as IL-1β, IL-6, and TNF-α, contributing significantly to the inflammatory response (131). Furthermore, the Toll-like receptor (TLR) pathway also triggers NF-κB activation, regulating the expression of multiple inflammatory cytokines (132). NF-κB serves as a regulatory hub for numerous inflammatory genes, amplifying monocyte chemoattractant protein-1 (MCP-1) expression, particularly in renal mesangial and tubular endothelial cells.

Several medicinal herbs have shown the ability to modulate NF-κB signaling and thus reduce inflammation. For instance, *Dioscorea opposita* Thunb. (Dioscoreaceae) is a plant of the dioscoreaceae family, and its main active ingredient, dioscin has demonstrated anti-inflammatory effects by downregulating NF-κB in rat models of fructose-induced renal damage, supporting the use of yam as a dietary supplement for patients with renal injury (25). Similarly, one of the active ingredients in *Glycyrrhiza alalensis* X.Y. Li (Fabaceae), 18α-Glycyrrhetinic acid, has been shown to suppress NF-κB signaling in fructose-incubated human kidney-2 (HK2) cells and mouse models, highlighting its potential anti-inflammatory effects (30). Another *G. alalensis* compound, isoangustone A, inhibits NF-κB activation in high-glucose-induced human mesangial cells (HMCs), reducing levels of intercellular adhesion molecule-1 (ICAM-1) and MCP-1 (34). Active ingredients extracted from *Siraitia grosvenorii* (Swingle) C. Jeffrey ex A.M. Lu & Zhi Y. Zhang (Cucurbitaceae), *Siraitia grosvenorii* polysaccharide, suppressed the inflammation via the TLR4/NF-κB pathways in cadmium chloride (CdCl_2_)-induced DKD mice (43). Additionally, components of *Zingiber officinale* Roscoe (Zingiberaceae), such as zingerone and 6-Shogaol, have shown substantial anti-inflammatory effects by inhibiting NF-κB in diabetic nephropathy models (52, 56). *Lycium barbarum* polysaccharides and zeaxanthin in *Lycium barbarum* L. (Solanaceae) have also exhibited renoprotective properties through NF-κB inhibition in various DKD models (59, 60). The active ingredients in *P. ginseng*, ginsenoside Rh1, effectively inhibited inflammatory factors and NF-κB signaling in high-fat diet (HFD) and streptozotocin (STZ)-induced DKD mice (100). In addition to these specific compounds, various medicinal herbs, such as *Gardenia jasminoides* J. Ellis (Rubiaceae), *Morus alba* L. (Moraceae) (Leaf), *Platycodon grandiflorus* (Jacq.) A. DC. (Campanulaceae), *Pueraria lobata* (Willd.) Ohwi (Fabaceae), *Taraxacum mongolicum* Hand. - Mazz. (Asteraceae), and *Prunella vulgaris* L. (Lamiaceae), have demonstrated significant potential in modulating the NF-κB signaling pathway to mitigate inflammatory responses (65, 69, 85, 86, 95, 102).

#### 3.1.2 Regulating IL signaling pathway

ILs are a diverse group of cytokines involved in both systemic inflammation and immune regulation, with 38 known variants, ranging from IL-1 to IL-38, based on their order of discovery. These cytokines influence various cell types and play critical roles in the progression of both inflammatory (133, 134) and non-inflammatory renal diseases (135, 136).

In DKD, IL-1β and IL-6 act as key pro-inflammatory mediators, significantly contributing to the disease’s inflammatory processes. *In vitro* studies have demonstrated that these cytokines stimulate the production of endothelin-1, which is associated with vascular dysfunction (137). Our research has shown that treatment with certain Chinese medicinal herbs—such as *D. opposita*, *Cassia obtusifolia* L. (Fabaceae), *S. grosvenorii*, *Lonicera japonica* Thunb. (Caprifoliaceae), *Z. officinale*, *G. jasminoides*, *M. alba* (Leaf), *P. grandiflorus*, Herba Taraxaci, *P. ginseng*, and *L. barbarum*—resulted in reduced levels of IL-1β and IL-6. This suggests these herbs may have clinical benefits in mitigating inflammation and slowing DKD progression. Animal studies also underscore the importance of IL-6 in kidney damage. Mice with hypertension induced by a high-salt and angiotensin II-based diet showed reduced proteinuria and blood pressure when IL-6 was knocked out (138). Additionally, recent findings emphasize the therapeutic potential of interleukin-1 receptor antagonist (IL-1RA) in blocking IL-1α and IL-1β activity, which are central to inflammatory diseases (139). Targeting IL-6 or IL-1 receptor genes or utilizing IL-1RA, has shown promise in protecting kidney function across various experimental models.

IL-17A, another pro-inflammatory cytokine, is associated with worsening kidney injury (140). Studies involving the active component luteolin, found in *L. japonica*, have demonstrated that it can suppress IL-17A secretion by inhibiting the signal transducer and activator of transcription (STAT) 3 pathway, producing anti-inflammatory effects in db/db mice (47). Reducing IL-17A may therefore offer a potential therapeutic strategy for DKD treatment (141). IL-18 has emerged as a reliable early marker for predicting delayed graft function after kidney transplantation (142, 143). Moreover, the active ingredients in Licorice, 18α-Glycyrrhetinic acid, has shown anti-inflammatory effects by reducing IL-18 in fructose (FRU)-incubated HK2 cells and FRU-induced mice (30). Anti-inflammatory cytokines, such as IL-4 and IL-10, also play vital roles in DKD prevention and treatment. For example, *P. lobata* and its active component puerarin have been shown to increase IL-4 expression, improving renal function in STZ-induced diabetic rats (87). Isoliquiritigenin, found in Licorice, displayed antioxidant effects and increased IL-10 secretion in STZ-induced DKD rats, suggesting its potential as a dietary supplement to impede DKD progression (31). Ethanol extracts of *L. japonica* were also found to elevate IL-10 in STZ-induced diabetic nephropathy rats, effectively mitigating DKD-related inflammation (46).

#### 3.1.3 Targeting the TNF-α signaling pathway

TNF-α is a potent pro-inflammatory cytokine that plays a central role in the pathogenesis of DKD (144). Diabetic patients, especially those with elevated urinary microalbumin levels, often exhibit serum TNF-α concentrations 3–4 times higher than non-diabetic individuals, positioning TNF-α as a potential early biomarker for DKD (145). TNF-α contributes to DKD progression by increasing mononuclear macrophage recruitment, reducing glomerular filtration rates, and altering endothelial permeability through hemodynamic changes. Additionally, it promotes the generation of ROS, damaging podocytes and endothelial cells, and leading to proteinuria and tubulointerstitial fibrosis (146).

Various natural herbs have demonstrated TNF-α inhibition and offer therapeutic potential in DKD. Components from *G. alalensis* (30, 31), *C. obtusifolia* (40), and *Vigna umbellata* (Thunb.) Ohwi & H. Ohashi (Fabaceae) (41) have shown TNF-α suppression alongside modulation of other inflammatory markers. Active ingredients from *Z. officinale*, such as zingerone (51, 52), zingiber officinale (53), 6-Gingerol (55), and zerumbone (57), have exhibited renoprotective effects by reducing TNF-α levels in STZ-induced DKD rats, highlighting *Z. officinale*’s therapeutic potential in managing DKD-related inflammation.

The active ingredients in *G. jasminoides*, genipin-1-β-d-gentiobioside, also demonstrated anti-inflammatory activity by reducing TNF-α production through the APMK/Sirt1/NF-κB pathway in STZ-induced DKD mice (65). *T. mongolicum* is favored by the Chinese as a heat-clearing and fire-reducing drink. Dandelion sterol was found to reverse TNF-α elevation in STZ-induced DKD rats and high glucose (HG)-incubated HK2 cells (95). Similarly, the primary component of *P. grandiflorus*, platycodin D, significantly reduced TNF-α and IL-1β levels while ameliorating renal cell apoptosis in HFD and STZ-induced DKD mice and HG-induced RAW264.7 cells (85). Other natural substances such as *D. opposita*, *Crataegus pinnatifida* Bunge (Rosaceae), *S. grosvenorii*, *L. japonica*, *L. barbarum*, *Morus alba* L. (Moraceae) (Fruit) and *P. ginseng* have also shown TNF-α regulation, supporting their potential in reducing inflammation and preserving kidney function.

#### 3.1.4 Targeting chemokines

Chemokines, small signaling proteins with conserved cysteine residues, are classified into four subfamilies: CC, CXC, CX3C, and C4, based on the arrangement of the first two cysteines. MCP-1, a member of the CC subfamily, is crucial for recruiting macrophages and mediating the inflammatory response.

Research indicates that Loganin, a water-soluble compound from *Cornus officinalis*, attenuates kidney injury in DKD by inhibiting macrophage infiltration via the MCP-1/chemokine (C-C motif) receptor 2 (CCR2) signaling pathways (147). Similarly, blocking the MCP-1/CCR2 axis using a CCR2 antagonist ameliorated DKD by reducing blood glucose levels, macrophage infiltration, and oxidative stress (148). Targeting MCP-1 expression is a promising strategy to limit macrophage recruitment and inflammation, thus delaying DKD progression.

*C. pinnatifida*, a fruit in the rosaceae family, showcases nutritional and medicinal value. Hyperoside, derived from *C. pinnatifida*, has shown reduced MCP-1 levels in HG-induced podocytes and mesangial cells, as well as in STZ-induced DKD rats, providing a basis for its use in DKD treatment (28). 18α-Glycyrrhetinic acid from *G. alalensis* also decreased MCP-1 and chemokine (C-C motif) ligand 3 (CCL3) levels, attenuating renal inflammation in DKD (30). *V. umbellata* is also one of the common beans as a traditional Chinese herb with decongestive effects. Studies have shown that Natto-red beans extract (41) and azuki bean seed coats (42) exhibited inhibition of MCP-1 in STZ-induced DKD rats, which fully demonstrated the main target to exert anti-inflammatory effects. In addition, *Z. officinale* (56, 57), *L. barbarum* (61), and *P. vulgaris* (102) have shown anti-inflammatory effects through MCP-1 reduction, supporting their role in managing DKD-related inflammation.

#### 3.1.5 Targeting adhesion molecules

Cell adhesion molecules, such as vascular cell adhesion molecule-1 (VCAM-1) and ICAM-1, are critical proteins expressed by endothelial cells and play an essential role in the binding of inflammatory cells. These molecules have been strongly implicated in the progression of DKD. Studies consistently report elevated levels of ICAM-1 and VCAM-1 in patients with DKD, with a significant correlation to microalbuminuria in type 2 diabetes patients compared to non-diabetic controls (149–151). Given the global emphasis on early detection of renal impairment and cardiovascular disease in patients with type 2 diabetes, soluble adhesion molecules have potential as biomarkers for vascular disease and early-stage DKD (152).

Fructus Mori, known for its anthocyanins, multivitamins, and niacin content, has shown therapeutic potential in DKD. Research by Abouzed et al. (67) demonstrated that black mulberry fruit extract alleviated VCAM-1 and TNF-α expression in STZ-induced DKD rats, suggesting its utility as a treatment option. In addition, a compound from Licorice, isoangustone A, has shown inhibitory effects on ICAM-1 expression in HG-induced HMCs (34). Similarly, zerumbone from *Z. officinale* has been found to reverse elevated ICAM-1 levels in STZ-induced DKD rats (57). A key component of *L. barbarum*, *Lycium barbarum* Polysaccharides, exhibit nephroprotective effects by reducing ICAM-1 levels and significantly inhibiting albuminuria in HFD and STZ-induced DKD mice. Furthermore, Puerarin, retrieved from *P. lobata* when administered at various doses, significantly reduced ICAM-1 levels in STZ-induced DKD mice, as confirmed by immunohistochemistry (88). Finally, the compound of *P. vulgaris* has shown promise in DKD treatment by inhibiting ICAM-1 expression, thereby reducing inflammation in HG-induced HMCs and STZ-induced diabetic rats.

#### 3.1.6 Adjusting TGF-β1/Smad signaling pathway

Several studies have identified the TGF-β1 signaling pathway and its downstream effectors, the Smad proteins, as key contributors to the development and progression of DKD. Elevated serum TGF-β1 levels are consistently observed in diabetic patients, making this cytokine a potential clinical indicator for DKD diagnosis (153). The Smad family of proteins, particularly Smad2/3/4/7, plays a significant role in DKD pathogenesis. Smad2/3, in particular, is activated in renal tissues of DKD patients, where it can exert either pro- or anti-inflammatory effects, depending on the context.

In models of HG-induced renal fibrosis and inflammation, the active ingredient of Licorice, isoangustone A, has demonstrated the ability to reverse mesenchymal transition via modulation of the TGF-β1/Smad signaling pathways when used at concentrations ≥ 10umol/L (34). Similarly, the active ingredient of *Z. officinale*, zingerone, has been shown to inhibit NF-κB activity and downregulate TGF-β expression, further supporting its anti-inflammatory role in DKD (154). Additionally, a randomized controlled trial found that turmeric, a member of the *Z. officinale* family, reduced TGF-β levels in patients with type 2 diabetic nephropathy, suggesting its potential therapeutic application (52). *L. japonica*, another herbal remedy, has also demonstrated significant anti-inflammatory properties. Ethanol extracts of *L. japonica* were found to reduce TGF-β1 expression in STZ-induced DKD rats, further indicating its potential as a therapeutic agent for DKD (46).

### 3.2 Anti-oxidative stress

**Figure 3:**
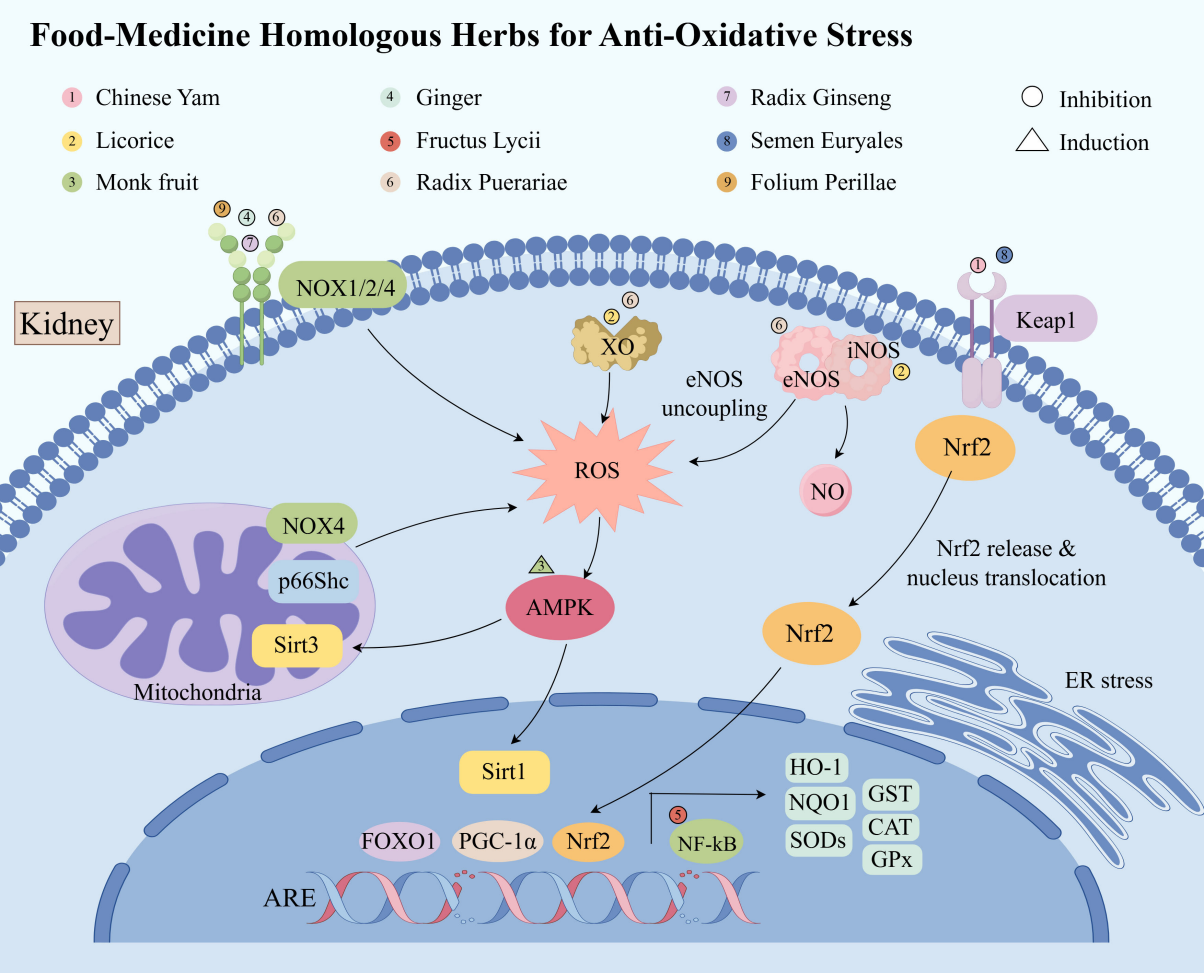
Food-medicine homologous herbs protect against DKD by alleviating oxidative stress.

Oxidative stress results from an imbalance between the overproduction of ROS and the reduced activity of endogenous antioxidants. This imbalance is implicated in the pathogenesis and progression of diabetes and its associated complications (155). Targeting oxidative stress through therapeutic interventions holds significant promise for the treatment of DKD (156). While antioxidant supplementation, such as vitamin C and E or superoxide dismutase (SOD)-mimicking compounds, has been explored in managing oxidative stress in DKD patients, these strategies have shown limited clinical benefit (157). In addition, pharmacological interventions aimed at modulating ROS sources and redox processes are under investigation in clinical trials; however, no definitive conclusions have yet been drawn (156). In this context, we shift our focus to herbs with anti-oxidative stress properties, which may offer a viable alternative for alleviating oxidative stress in DKD. These herbs exhibit the ability to inhibit ROS generation, either from mitochondrial or non-mitochondrial sources, and modulate redox pathways. As such, they could serve as dietary antioxidant supplements for patients with DKD (**Figure 3**).

#### 3.2.1 Reducing mitochondrial ROS sources

Reducing ROS generation is a crucial strategy for mitigating oxidative stress. Mitochondria are primary sources of ROS. Key mitochondrial ROS-producing sites include the electron transport chain, nicotinamide adenine nucleotide phosphate oxidase (NOX), p66Shc, monoaminoxidase (MAO), α-Glycerophosphate dehydrogenase, electron transfer flavoprotein (ETF), ETF quinone oxidoreductase (ETF dehydrogenase), and aconitase (158). Seven isoforms of NOX (NOX1–5 and DUOX1–2) have been identified, each residing in different specialized tissues and intracellular locations. Among these, NOX4 is unique in its mitochondrial localization. The specific roles of the various NOX subunits in kidney injury are still debated (159). NOX1, NOX2, and NOX4 have been implicated in high glucose-dependent oxidative stress, with NOX4 being considered a major ROS source in DKD and a key player in the renal oxidative stress pathway (160–162). Additionally, studies indicate that ROS generated by p66Shc regulate the expression and activity of NOX4, thereby amplifying oxidative stress. p66Shc may serve as a biomarker for renal oxidative injury in DKD patients (163, 164). In DKD mice, NOX1, NOX2, NOX4, and p66Shc levels were significantly elevated in renal tissues, and inhibiting NOX and p66Shc expression offered protection against DKD development (165, 166).

The component of *Z. officinale*, zingerone, has been reported to attenuate the oxidative stress in kidneys of db/db mice by downreguating NOX4 (51). Extracts from *Perilla frutescens* (L.) Britton (Lamiaceae) sprouts inhibit ROS overproduction in HG-medicated glomerular mesangial cells by suppressing NOX2 and NOX4 (110). An active compound from *P. lobata*, puerarin, has been shown to attenuate diabetic kidney injury by suppressing NOX4 expression in podocytes (167). The active ingredients in *P. ginseng*, ginsenoside Rh1 reduces kidney damage by inhibiting NOX1 and NOX4 protein expression in HFD/STZ induced DKD mice (100). Furthermore, a study revealed that 250mg/kg *D. opposita*’s active ingredient, dioscorea zingiberensis, can reduce p66Shc expression in the kidneys, leading to increased levels of antioxidant enzymes such as SOD and catalase (CAT) (26).

#### 3.2.2 Reducing non-mitochondrial ROS sources

In addition to mitochondrial sources, ROS can also be generated from non-mitochondrial sources such as endothelial nitric oxide synthase (NOS) and xanthine oxidase (XO). NOS produces nitric oxide (NO), which is divided into three isoforms: endothelial nitric oxide synthase (eNOS), neuronal nitric oxide synthase (nNOS) and inducible nitric oxide synthase (iNOS). Studies on NOS and NO levels in diabetes have yielded conflicting results. Xu et al. (168) propose that this discrepancy may be due to “eNOS uncoupling”, a phenomenon where eNOS produces superoxide (O_2_) instead of NO. In several *in vitro* and animal models of cardiovascular disease, as well as in patients with cardiovascular risk factors, eNOS has been observed to shift from a protective role to one that contributes to oxidative stress (169). XO, when abnormally activated by hyperglycemia, increases intracellular ROS levels, thereby exacerbating oxidative stress-induced kidney injury (170).

A bioactive component of Licorice, 18α-Glycyrrhetinic acid, has been reported to reduce oxidative stress markers or related products such as ROS and H_2_O_2_ in fructose-induced HK2 cells and in kidneys of fructose-challenged mice, which may be related to its modulation of iNOS and XO activities (30). *P. lobata*’s active component, puerarin, can improve renal function by attenuating eNOS expression in glomerular endothelial cells and tubular cells of STZ-induced diabetic rats (91).

#### 3.2.3 Regulating the Keap1/Nrf2/ARE signaling pathways

The Keap1/Nrf2/ARE pathway is a crucial antioxidant signaling mechanism involved in the cellular antioxidant defense system. Nuclear factor erythroid 2-related factor 2 (Nrf2) regulates the expression of antioxidant response elements (ARE) and plays a key role in maintaining cellular homeostasis. Kelch-like ECH-associated protein 1 (Keap1) is a major inhibitor of Nrf2. Under normal conditions, Nrf2 binds to Keap1 and is subsequently ubiquitinated and degraded by the proteasome. When Keap1 activity is inhibited, Nrf2 accumulates and translocates to the nucleus, where it binds to ARE and induces the expression of various enzymatic antioxidants, including heme oxygenase 1 (HO-1), NADPH: quinone oxidoreductase-1 (NQO1), CAT, total superoxide dismutase (T-SOD), glutathione peroxidases (GPx), glucagon-like peptide peroxidase (GSH-Px) and so on (171, 172).

In DKD mice models, Keap1 was overactivated, resulting in decreased levels of Nrf2 and enzymatic antioxidants. *D. opposita*’s active ingredient, dioscin, has been reported to alleviate oxidative stress by decreasing Keap1 expression and increasing Nrf2 and glutathione S-transferase (GST) levels (25). *Euryale ferox* Salisb. ex K.D. Koenig & Sims (Nymphaeaceae) has also reported to improve oxidative stress by regulating the Keap1/Nrf2/HO-1 pathways (106). Additionally, the active ingredients of Licorice, 18α-Glycyrrhetinic acid (30) and licochalcone A (35), a major biologically active components extracted from *Z. officinale*, 6-Shogaolone (56), and the leaf extract of *Nelumbo nucifera* Gaertn. (Nelumbonaceae) (108) can suppress oxidative stress in DKD mice models by stimulating the Nrf2 signaling pathway.

#### 3.2.4 Activating the AMPK/Sirt signaling pathways

AMP-activated protein kinase (AMPK) is a key upstream regulator of the antioxidant response, and its activation inhibits oxidative stress associated with various pathologies. Sirtuins, a family of proteins that includes Sirt1-Sirt7, play a significant role in this process. Sirt1, the most extensively studied sirtuin, works in conjunction with AMPK to activate antioxidant signaling through several downstream effectors, such as peroxisome proliferator-activated receptor-gamma coactivator-1alpha (PGC-1α) and the forkhead box O (FOXO) transcription factor, which helps mitigate oxidative stress (173, 174). Sirt1 reduces the acetylation of FOXO1, enhancing its DNA-binding affinity and thereby increasing the expression of manganese superoxide dismutase (Mn-SOD) and CAT (175). Moreover, Sirt1 significantly boosts the activity of the Keap1/Nrf2/ARE pathway by decreasing Keap1 expression and promoting the Nrf2’s nuclear accumulation, ARE-binding capacity, and transcriptional activity (176). Sirt3, another sirtuin, has been shown to have antioxidant effects by enhancing the deacetylation and activation of isocitrate dehydrogenase 2 (IDH2) and the glutathione antioxidant defense system (177). Thus, activating the AMPK/Sirt signaling pathway to reduce hyperglycemia-induced oxidative stress may help prevent the progression of DKD.

A principal active component from *S. grosvenorii*, mogroside IIIE, has been reported to alleviate HG-induced oxidative stress by activating the AMPK/Sirt1 signaling pathway (44). *E. ferox* can improve oxidative stress according to activate the AMPK/mammalian target of rapamycin (mTOR) pathway in DKD mice (106). *P. frutescens*’s sprout extract and the major active substances of ginseng, ginsenoside Rh1 can alleviate DKD via increasing AMPK expression in STZ/HFD-induced DKD mice and HG-medicated glomerular mesangial cells (100, 110). *P. lobata*’s active component, puerarin, can significantly upregulates Sirt1, FOXO1 and PGC-1α expressions in the renal cortex, potentially offering protection against DKD by attenuating oxidative stress (86). A bioactive flavonoid from Licorice, isoliquiritigenin, protects against DKD rats by increasing the expression of Sirt1 (31). *D. opposita*’s active ingredient, dioscin, also shows protective effects against FRU-induced renal damage by modulating Sirt3-mediated oxidative stress (25).

#### 3.2.5 Inhibiting the NF-κB signaling pathway

ROS interact with the NF-κB signaling pathway in several ways. Firstly, NF-κB activity is mainly regulated by IκB proteins. For instance, the IκB inhibitor BAY 11–7082 has been found to reduce NF-κB activation, thereby alleviating oxidative stress in diabetic rat models (178). Conversely, ROS can activate NF-κB by inducing the phosphorylation of IκBα (179). Additionally, it has been reported that *L. barbarum*’s active ingredient, lycium barbarum polysaccharide, can decrease the expression of IκBα and reduce NF-κB activity in kidney tissues of STZ-induced diabetic rats (59).

### 3.3 Anti-fibrosis

Renal fibrosis is a crucial pathological process in the progression of DKD to end-stage renal disease (180, 181). Renal fibrosis is marked by the activation of α-smooth muscle actin (αSMA)-positive myofibroblasts under pathological conditions, leading to excessive accumulation and deposition of extracellular matrix (ECM) proteins (182, 183). Emerging evidence indicates that epithelial-to-mesenchymal transition (EMT) is a key mechanism in the development of renal fibrosis. During EMT, renal tubular epithelial cells (TECs) lose their epithelial characteristics and adopt a mesenchymal phenotype, contributing to the formation of intermediate stromal myofibroblasts (184, 185). TECs begin to express fibroblast markers and lose their epithelial identity, resulting in ECM remodeling and the progression of renal fibrosis (186).

**Figure 4:**
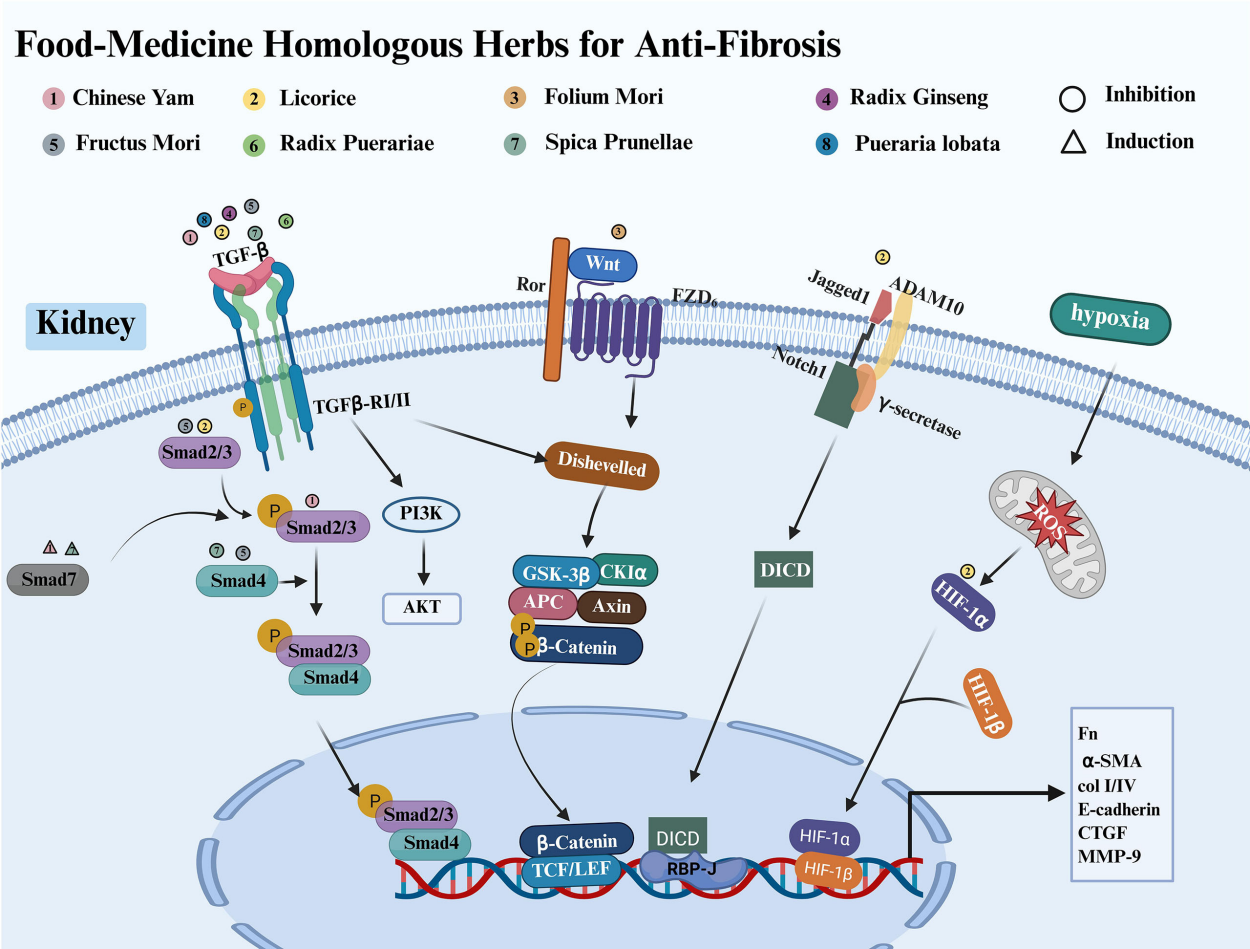
Food-medicine homologous herbs protect against DKD by inhibiting kidney fibrosis.

Renal fibrosis damages the renal parenchyma and impairs renal function, closely influencing the progression and prognosis of DKD. Therefore, targeting renal fibrosis is essential for the treatment of DKD. Currently, there are no specific treatments designed exclusively to inhibit renal fibrosis. However, there is substantial evidence supporting the use of “herbs as food” as a natural approach to mitigate renal fibrosis through various mechanisms and signaling pathways. This section will focus on the mechanisms and signaling pathways through which herbs as food can inhibit renal fibrosis (**Figure 4**).

#### 3.3.1 Target regulation of fibrotic markers

Excessive production and deposition of ECM components are hallmarks of renal fibrosis. Key ECM markers for interstitial fibrosis include collagen type I (Coll І), collagen type III (Coll III), collagen type IV (Coll IV), fibronectin (FN) and laminin (187, 188). MCP-1 is also implicated in renal tubulointerstitial fibrosis and serves as a fibrosis marker (189, 190). Various herbs have been shown to target these fibrosis-related proteins, offering potential therapeutic benefits for DKD.

Studies have demonstrated that the water-soluble extract of *Cinnamomum cassia* (L.) J. Presl (Lauraceae) reduces overproduction of FN and Coll IV in HG-induced mesangial cells, thereby inhibiting renal fibrosis (105). Active components of *Z. officinale*, including zingerone and 6-shogaol, have been found to mitigate fibrosis in db/db mice by decreasing FN and Coll IV expression (51, 56). Additionally, an active ingredient of *Z. officinale*, zerumbone, down-regulates MCP-1, reduces FN deposition in the ECM, and protects renal function in rats with STZ-induced diabetic nephropathy (57). *V. umbellata* seed coat polyphenols have been reported to inhibit MCP-1 messenger RNA (mRNA) expression and alleviate renal fibrosis in STZ-induced diabetic rats (42). Other herbs, such as *P. frutescens*, *M. alba* (Leaf), and *L. japonica*, have also shown the ability to inhibit fibrosis in DKD models by targeting and modulating fibrosis marker proteins like FN and Coll IV (47, 67, 110).

#### 3.3.2 Regulating the TGF-β/Smad signaling pathway

The development of DKD involves various signaling pathways, with the TGF-β/Smad signaling pathway playing a pivotal role. Among the three isoforms of TGF-β: TGF-β1, TGF-β2, and TGF-β3. TGF-β1 is most highly expressed in the kidney and is crucial in renal fibrosis (191). Smad proteins are significant effector molecules downstream in the classical transduction pathway of TGF-β1 signaling. TGF-β1 initiates pro-fibrotic effects by binding to its receptors, leading to the recruitment and activation of Smad2/3 proteins. The activated Smad2/3 complex then translocates to the nucleus to induce the transcription of genes associated with fibrosis (192, 193). In DKD, a high-glucose environment and advanced glycation end-products (AGEs) activate the TGF-β1/Smad signaling pathway. This activation inhibits matrix metalloproteinase (MMP) synthesis while promoting the production of tissue inhibitors of metalloproteinases (TIMPs). Consequently, ECM synthesis is increased, leading to excessive ECM accumulation and fibrosis (186, 194). Therefore, targeting the TGF-β1/Smad pathway is a promising strategy for combating renal fibrosis in DKD. Several herbs have demonstrated potential in modulating the TGF-β1/Smad signaling pathway.

*D. opposita*, contains dioscin, which down-regulates TGF-β1 and phosphorylated Smad3 (p-Smad3), and up-regulates Smad7 in a fructose-induced kidney injury model, exerting anti-fibrotic effects (99). *G. alalensis* extract protects against HG-induced renal damage by inhibiting the protein kinase C beta 2 (PKCb2)-dependent TGF-β1/Smad signaling pathway (37). This extract also inhibits TGF-β1 expression in HG-induced HK-2 renal proximal tubular epithelial cells and HG diet-induced type 2 diabetes mellitus Apoe^em1/Nar1^/Narl mice model, thereby down-regulating downstream Smad3 signaling and reducing connective tissue growth factor (CTGF) and Coll I levels, which mitigates renal fibrosis (38, 39). Furthermore, *G. alalensis* also contains active components such as isoliquiritigenin and isoangustone A, which further inhibit the TGF-β1/Smad signaling pathway and reduce glomerular matrix accumulation in a HG environment (33, 34). Additionally, *M. alba* (Fruit) extracts, which include flavonoids, polysaccharides, and alkaloids, have been shown to down-regulate TGF-β1, Smad2, Smad3, Smad4, and CTGF, thereby exerting anti-fibrotic effects in db/db mice and STZ-injected male C57BL/6 mice (77, 79). Ji T et al. (80) discovered that the combination of *M. alba* (Leaf) alkaloids and flavonoids extract synergistically modulates the TGF-β1/Smad pathway to alleviate DKD. The main active component of Fructus Mori, *M. alba* (Fruit) alkaloids, ameliorates DKD by inhibiting the Zucker diabetic fatty rats TGF-β1 signaling pathway. Pueraria mirifica, derived from the dried root of *P. lobata* (76), contains puerarin, which has been reported to exert anti-fibrotic effects in STZ-induced diabetic rats by down-regulating Coll IV, FN, and other pro-fibrotic growth factors. This effect may be associated with the inhibition of the TGF-β1/Smad2 pathway (87, 90, 94). Ginsenoside Rg1 ameliorates glomerular fibrosis in type 2 diabetic mice by inhibiting the RPC2/NFAT2 signaling-dependent TGF-β1/Smad2/3 pathways (99). Aqueous extract of *P. vulgaris* block the TGF-β1/Smad signaling pathway, reduces the expression of TGF-β and Smad2/4, increases Smad7 levels, thereby inhibiting the formation of the fibrosis markers CTGF and collagen IV and ameliorating renal fibrosis in STZ-induced diabetic rats (102).

#### 3.3.3 Regulating other signaling pathways

The Wnt/β-catenin signaling pathway, like TGF-β, is activated under high glucose conditions and regulates downstream target genes such as fibroblasts, Snail1, and MMP-7. These genes are involved in apoptosis, EMT, and renal tubular dysfunction, which contribute to renal and interstitial fibrosis (195, 196). Recent studies have shown that a combination of mulberry leaf alkaloids and flavonoids can ameliorate renal fibrosis in high glucose-induced diabetic SD rats by targeting and modulating the Wnt/β-catenin signaling pathway. Notably, this pathway works synergistically with the TGF-β/Smad signaling pathway (80).

The Notch signaling pathway, crucial for determining cell fate, includes receptors (Notch1-4), ligands (Delta-like1,2,3, Jagged1, and Jagged2), and various downstream signaling molecules. Evidence indicates that the Notch signaling pathway is essential in regulating renal epithelial-mesenchymal transition and fibrosis in DKD (197). *G. alalensis* extracts protect rat kidney cells by blocking Notch2 signaling activated by high glucose. This effect is mediated through down-regulation of Delta-1 and Jagged1 and suppression of downstream target genes involved in renal tubular EMT and fibrosiss (39).

The extracellular regulated protein kinases (ERK) signaling pathway, activated by high glucose, catalyzes the phosphorylation of downstream products such as TGF-β. This process induces EMT and contributes to fibrosis formation (198, 199). Tao Ji et al. (70) utilized a network pharmacological approach to predict anti-renal tubulointerstitial fibrosis effects via the ERK1/2 signaling pathway, a finding later confirmed in human renal tubular epithelial cells induced by high glucose.

Hypoxia inducible factor-1α (HIF-1α), a transcriptional activator, is closely linked to oxygen levels in the body. Prolonged high glucose exposure in DKD patients increases oxidative stress and microvascular dysfunction, leading to renal hypoxia due to inadequate oxygen supply (200). Under hypoxic conditions, HIF-1α translocates to the nucleus, regulates downstream proteins, promotes ECM deposition, and accelerates renal fibrosis progression (201–203). An active component of Licorice, licochalcone A, inhibits HIF-1α, TGF-β1 and AGEs, thereby ameliorating renal fibrosis in STZ-induced type 2 diabetic mice (33).

The sphingosine kinase 1-sphingosine 1-phosphate (SphK1-S1P) signaling pathway plays a role in DKD progression (204). HG, AGE, and oxidative stress stimulate SphK1 to produce S1P, which acts as an intracellular second messenger. S1P activates TGF-β, mimics TGF-β-induced cellular responses, and accelerates renal fibrosis (205). Curcumin, a polyphenolic compound found in *Curcuma Longa* L. (Zingiberaceae), has been reported to down-regulate SphK1 and S1P expression, inhibit FN and TGF-β1 production mediated by the SphK1-S1P signaling pathway, and exert nephroprotective effects by ameliorating DKD fibrosis (103, 104).

### 3.4 Food-medicine homologous herbs for modulation of other targets

**Figure 5:**
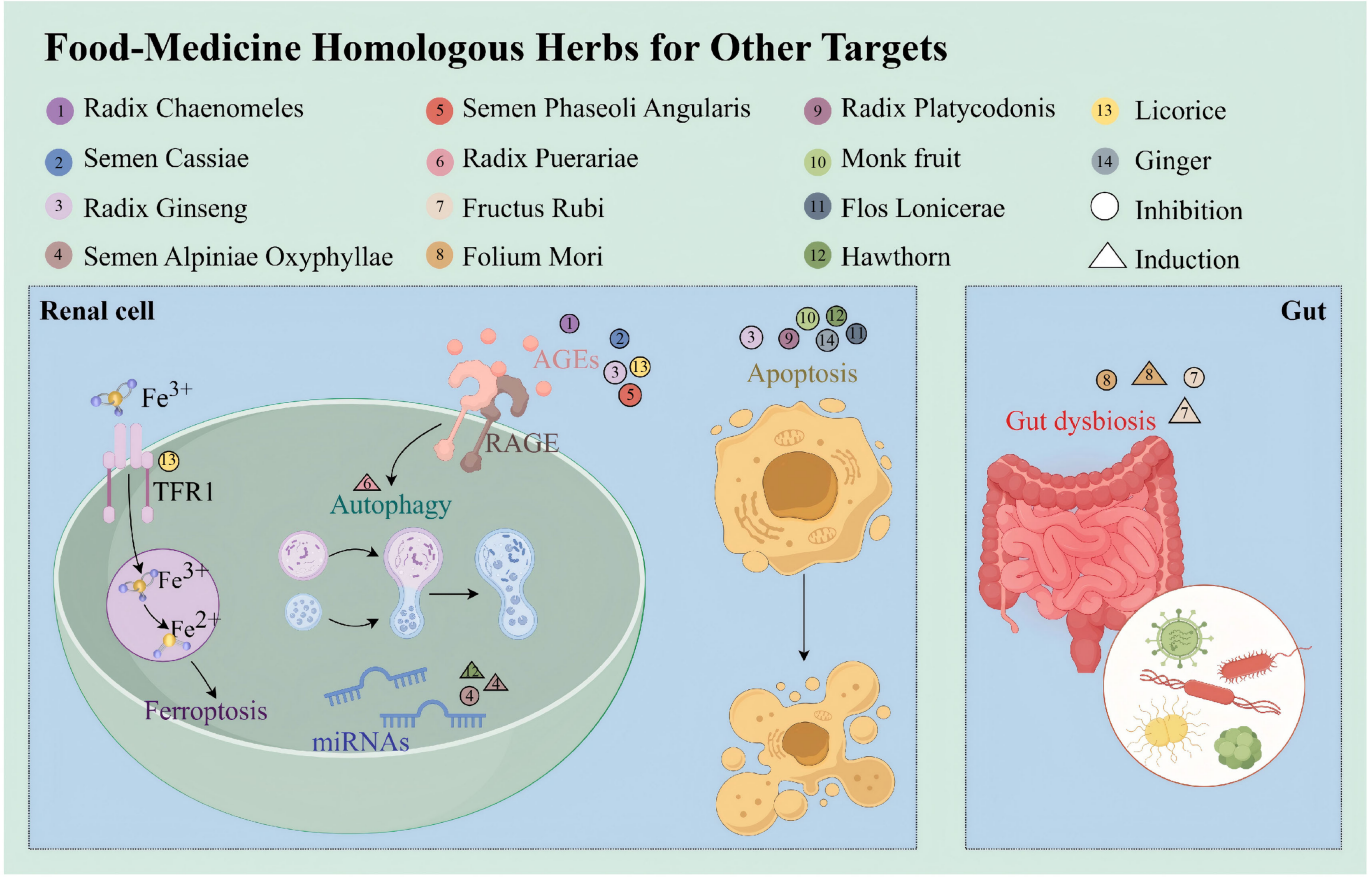
Food-medicine homologous herbs protect against DKD by modulating other targets.

In the pathogenesis of DKD, inflammation, oxidative stress, and fibrosis represent the most critical pathological mechanisms. Beyond these, other key processes include pathological alterations that determine renal cell fate, such as apoptosis, autophagy, and ferroptosis, as well as extra-renal mechanisms like gut dysbiosis. Food-medicine homologous herbs have demonstrated potential in ameliorating these pathological processes (**Figure 5**).

#### 3.4.1 Inhibiting AGEs/RAGE axis

AGEs are primarily formed through the Maillard reaction, which is significantly accelerated under persistent hyperglycemic conditions (206). AGEs contribute to diabetes pathophysiology mainly through cross-link formation and binding to their receptor, the receptor for advanced glycation end products (RAGE). This binding activates various oxidative stress and inflammatory signaling pathways, including TGF-β and NF-κB (207). Consequently, AGEs are considered critical in the progression of diabetes and its complications. Thus, targeting the AGEs/RAGE axis through pharmacological interventions may offer an effective therapeutic approach.

Recent studies have highlighted that certain medicinal herbs can inhibit the AGEs/RAGE axis, potentially slowing the progression of diabetes-related complications. For instance, *Chaenomeles sinensis* (Thouin) Koehne (Rosaceae Juss), rich in polyphenols, has been shown to inhibit the formation of α-dicarbonyl compounds, which are precursors to AGEs, in diabetic KK-A(y) mice (114). A bioactive component of Licorice, licochalcone A, has been reported to reduce kidney fibrosis in STZ-induced DKD mice by inhibiting the AGEs/RAGE axis (35). Additionally, *V. umbellata* and the active ingredients in *P. ginseng* (ginsenoside Rh1 and 20(R)-ginsenoside Rg3) have been found to effectively prevent excessive AGEs production in STZ-induced DKD mice (41, 100, 101). Moreover, Cassiae Semen extract has demonstrated renal protective effects by down-regulating RAGE expression in STZ-induced DKD rats (40).

#### 3.4.2 Regulating gut dysbiosis

Gut dysbiosis refers to a disruption in the composition, diversity, and function of the gut microbiota, as well as changes in its metabolite profile, which can subsequently affect the serum metabolome (208). Research indicates that gut dysbiosis plays a role in the onset and progression of diabetes and its complications through complex and not fully understood mechanisms. Therefore, modulating gut dysbiosis has emerged as a promising therapeutic approach (209).

Certain medicinal herbs may help slow the progression of diabetes and related complications by regulating gut dysbiosis. For example, *Rubus chingii* Hu (Rosaceae), which are rich in pelargonidin-3-O-glucoside, have been shown to modulate the gut microbiota in db/db mice. This modulation increases the abundance of *Prevotella* and *Lachnospiraceae* and enhances fecal short-chain fatty acids (SCFAs) content (96, 97). *M. alba* (Leaf) was also reported to alleviate STZ/HFD-induced DKD models by modulating the gut microbiota and increasing fecal SCFAs, moreover, it can regulate the serum and urine metabolome and increase fecal bile acids (68, 71–75, 80). An active ingredient from *S. grosvenorii*, *Siraitia grosvenorii* polysaccharide, has been found to improve and stabilize the serum metabolome of DKD mice, primarily by modulating 39 differentiated metabolites, including arachidonic acid (43).

#### 3.4.3 Attenuating apoptosis

Apoptosis is a regulated form of cell death characterized by the activation of apoptotic caspases, a group of cysteine-aspartyl proteases. Key members of this group include caspase-3, -6, -8, and -9, which cleave specific target proteins, leading to downstream effects such as DNA fragmentation (210). B-cell lymphoma-2 (Bcl-2) family proteins are crucial regulators of mitochondrial apoptosis, functioning as either promoters or inhibitors of apoptosis. Anti-apoptotic Bcl-2 family members (e.g., Bcl-2 and Bcl-XL) inhibit apoptosis, while pro-apoptotic members (e.g., Bax and BAK) promote it (211). The regulation of apoptosis is also influenced by the phosphatidylinositol 3-kinase (PI3K)/protein kinase B (AKT)/AMPK signaling pathway (212). In STZ-induced DKD mice, it has been observed that PI3K/AKT activity is significantly elevated, whereas AMPK activity is markedly reduced, thereby promoting apoptosis (213, 214).

Several medicinal herbs have been shown to modulate apoptosis through these signaling pathways. A key component of *P. grandiflorus*, platycodin D, and the active ingredients in *P. ginseng*, ginsenoside Rh1 have been reported to significantly reduce the expression of Bax, cleaved-caspase-3, and cleaved-caspase-9, while increasing the expression of Bcl-2 and Bcl-XL in the kidneys of STZ-induced DKD mice. This modulation occurs through the PI3K/AKT/AMPK-mediated apoptosis signaling pathway (85, 100). The principal active component from *S. grosvenorii*, mogroside IIIE, has been found to alleviate HG-induced apoptosis of podocytes by activating the AMPK/Sirt1 signaling pathway (44). Additionally, the active ingredients in *P. ginseng*, 20(R)-ginsenoside Rg3 enhances the expression of Bcl-2 and Bcl-XL and decreases the levels of Bax, cleaved-caspase-3, and caspase-8 in STZ-induced DKD mice (101). An active ingredient of *L. japonica*, luteolin, attenuates apoptosis by increasing Bcl-2 expression and decreasing the level of cleaved caspases -3, -6 and -9 in HG-treated mouse podocyte cell-5 (MPC-5) cells (48).

#### 3.4.4 Activating autophagy

Autophagy and apoptosis interact closely with each other. Autophagy is a process that removes damaged proteins and organelles to maintain intracellular homeostasis (215). This mechanism involves several multiprotein complexes that regulate each step, with AMPK playing a key role in this regulation (216). Sustained endoplasmic reticulum stress activates the protein kinase R-like ER kinase-eukaryotic translation initiation factor 2 alpha (PERK-eIF2α) signaling pathway. This activation leads to the upregulation of activating transcription factor 4 (ATF4) through eIF2α phosphorylation, followed by the activation of CHOP and Beclin-1, which initiate an autophagic response (217, 218). An active compound from *P. lobata*, puerarin, has been shown to activate autophagy and alleviate DKD in both STZ-induced DKD mice and HG-induced podocytes. It achieves this by activating the AMPK signaling pathway, increasing the protein levels of Sirt1 and LC3B, decreasing p62 levels, and reversing the acetylation of liver kinase B1 (LKB1) (92). Additionally, puerarin promotes autophagy and protects against kidney damage in STZ-induced DKD mice by activating the PERK/eIF2α/ATF4 signaling pathway and upregulating the expression of autophagy markers Beclin-1 and Atg5 (93).

#### 3.4.5 Inhibiting ferroptosis

Ferroptosis is an iron-dependent form of cell death driven by lipid peroxidation, which plays a crucial role in the development and progression of diabetes and DKD (219). Key regulators of ferroptosis include System Xc, GPx4, and transferrin receptor 1 (TFR1) (220). System Xc is a disulfide-bonded heterodimer comprising a light chain subunit (SLC7A11) and a heavy chain subunit (SLC3A2), which is widely distributed in cell membranes (221). Emerging evidence suggests that herbs can modulate ferroptosis in renal cells and hold potential for improving DKD (222). Glabridin, a bioactive component of licorice, ameliorates DKD by regulating ferroptosis. It upregulates GPx4, SLC7A11, and SLC3A2 and downregulates kidney iron content and TFR1 in STZ/HFD-induced DKD rats and HG-induced NRK-52E cells (36).

#### 3.4.6 Targeting miRNAs

MicroRNAs (miRNAs) are small non-coding RNAs that regulate gene expression at the translational level. Altered miRNA expression profiles have been observed in both diabetes and DKD, and these differentially expressed miRNAs have potential as biomarkers and therapeutic targets (223). A natural compound extracted from the *C. pinnatifida*, hyperoside, alleviates HG-induced mesangial cell proliferation by inhibiting the ERK/CREB/miRNA-34a signaling pathway (29). Another study demonstrated that hyperoside could inhibit renal injury and fibrosis in STZ-induced DKD mice and HG-induced podocyte models by increasing miR-499e-5p levels (28). *Alpinia oxyphylla* Miq. (Zingiberaceae) alters miRNAs expression profiles, including miR-378d, miR-129-1-3p, miR-21a-5p, miR-29c-3p, miR-203-3p and miR-7a-5p, in db/db mouse kidneys (115).

## 4 Discussion

The pathology of DKD is complex, involving mechanisms such as inflammation, oxidative stress, fibrosis, apoptosis, autophagy, and ferroptosis. As interest in targeted therapies for kidney disease grows, the development of predictive DKD biomarkers has become a prominent research focus. However, current biomarkers often target specific mechanisms within the disease process. For instance, urinary and plasma KIM-1, neutrophil gelatinase-associated lipocalin (NGAL), and MCP-1 are strongly associated with renal tubular injury (224–226). Similarly, urinary and plasma 8-hydroxydeoxyguanosine (8-OHdG) are linked to oxidative stress (227, 228), while plasma TNF-α and tumor necrosis factor receptor (TNFR) are associated with inflammation (229, 230). Novel biomarkers based on proteomics (231, 232), metabolomics (233, 234) and transcriptomics (235, 236) have emerged, contributing to disease assessment, treatment monitoring, and prognosis. Despite their utility, single-mechanism biomarkers have limitations. The albumin creatinine ratio (ACR) and eGFR remain the most frequently used biomarkers for assessing treatment efficacy and risk in DKD patients (237). Future research may benefit from exploring combinations of multiple biomarkers to better predict DKD progression.

The treatment of DKD remains an open question, but the development of targeted therapeutic agents holds promise due to the disease’s complex pathomechanism. NF-κB, a core transcription factor, plays a crucial role in signaling pathways, inducing both inflammatory responses and oxidative stress (178, 238, 239). TGF-β1/Smad as a key signaling pathway in the pathogenesis of DKD, affects disease progression by inducing and promoting both inflammation and fibrosis due to its large and complex network of mechanisms, and DKD can be treated by rebalancing TGF-β1/Smad signaling (240–242). Additionally, the mitogen-activated protein kinase (MAPK) pathway, including p38 MAPK and c-Jun N-terminal kinase (JNK), mediates inflammation, oxidative stress, and fibrosis simultaneously (243, 244). Focusing on these upstream targets could enhance therapeutic strategies, as targeting multiple pathologic mechanisms simultaneously may offer more clinical benefits.

TCM demonstrates unique advantages in the prevention and treatment of DKD due to its multi-component, multi-target, and multi-pathway characteristics (245, 246). Tang Ge et al. (247) systematically reviewed the clinical efficacy, potential mechanisms, and molecular targets of TCM compounds and bioactive components in treating DKD. Meanwhile, Hu Q et al. (248) focused on natural product-based solutions for DKD, providing new insights for drug development, with many of these natural products originating from food-medicine homologous herbs. In contrast to the focus of the aforementioned studies, this paper centers on food-medicine homologous herbs, summarizing TCM recommendations that are more readily available daily, safer, and offer better palatability (**Table 1**). Many of these herbs have been included in the safety food lists of various countries, offering important references for the development of functional foods, dietary supplements, and daily dietary recommendations for DKD patients. Existing evidence suggests that phytonutrients and plant-based diets not only have potential benefits for the primary prevention of CKD but also help delay the progression in patients with CKD stages G3-5 (249). All 29 food-medicine homologous herbs summarized in this paper are plant-based. Incorporating them into the daily diet of DKD patients can effectively exert adjunctive therapeutic effects, providing an easily implementable and well-adhered-to dietary management strategy. Given that DKD is fundamentally a metabolic disorder, those food-medicine homologous herbs that have been demonstrated to ameliorate disordered lipid metabolism also warrant attention and could be considered for incorporation into patients’ daily dietary plans (250, 251). It should be noted that, beyond food-medicine homologous herbs, many traditional Chinese herbs, such as Astragali Radix (*Astragalus membranaceus* (Fisch.) Bge.), Scutellariae Radix (*Scutellaria baicalensis* Georgi.), classified solely for medicinal use also possess well-documented efficacy against DKD and have accumulated extensive clinical experience (245, 252–254). In comparison, food-medicine homologous herbs possess inherent advantages in long-term dietary safety, daily accessibility, and patient acceptability, making them more suitable for long-term use as dietary aids integrated into daily meals. In contrast, single herbs or active components may offer more potent pharmacological effects and target specificity, but the safety of some herbs during long-term or high-dose use requires further evaluation. Therefore, in clinical practice, the two categories can often complement each other, balancing safety and therapeutic efficacy.

Integrating TCM principles of xing (nature), wei (flavor), and gongxiao (efficacy) with contemporary nutritional science, this review categorizes commonly used food-medicine homologous herbs into five functional dietary groups: beverages, soups/porridges, seasonings, snacks, and specialized formulations. Specific examples and considerations for each group are detailed below: (1) Beverages: Herbal infusions offer readily accessible therapeutic benefits. *C. pinnatifida* (3 g), *C. obtusifolia* (3 g), and *N. nucifera* (3* g*) may promote lipid metabolism, although caution is advised for individuals with hyperchlorhydria. *L. japonica* (5 g), *T. mongolicum* (3* g*), and *G. alalensis* (2* g*) provide potential heat-clearing effects, enhanced by *Z. officinale* in cases of spleen-stomach deficiency. Finally, *M. alba* (Leaf) (3 g), *L. barbarum* (3 g), and *Mentha haplocalyx* Briq. (Lamiaceae) (2 slices) are traditionally used to support visual health. (2) Soups/Porridges: Hearty and nutritious, these formulations offer sustained benefits. A porridge combining *D. opposita* (50g), *E. ferox* (15 g), and *V. umbellata* (20* g*) aims to strengthen the spleen and resolve dampness. A paste of *P. lobata* (20 g) with *L. barbarum* (3 g) and *Sesamum indicum* L. (Pedaliaceae) (5 g) is traditionally employed for Yin nourishment. Furthermore, chicken soup infused with Radix Angelicae Ainensis (3 g), *Z. officinale* (3 slices), and *C. sinensis* (100* g*) is believed to warm the meridians and nourish blood, requiring careful consideration in individuals exhibiting Yin deficiency with fire. (3) Seasonings: Integrating herbs into daily cooking can provide subtle, yet consistent therapeutic benefits. *C. cassia* (1 g) and *P. cyrtonema* (1* g*) can be added to beverages to promote Yang warming and blood activation. *P. frutescens* enhances salads, while *P. grandiflorus* (3 g) serves as a dipping sauce with potential cold-preventative properties. (4) Snacks: Utilizing herbs as readily available snacks encourages consistent consumption *M. alba* (Fruit) and *R. chingii* (10 g daily) are consumed for liver-kidney tonification and antioxidant effects. *S. grosvenorii* are traditionally used to moisten the lungs. (5) Specialized Formulations: These targeted therapies are best administered under professional guidance. A decoction of *P. ginseng* (2 g) and *C. Longa* (5 g) may benefit individuals with Qi deficiency, although close monitoring is crucial in hypertensive patients. *G. jasminoides* (3 g) is used to promote calmness and enhance sleep. It is paramount to emphasize that herb selection should be individualized based on a comprehensive assessment of the patient’s constitutional pattern according to TCM principles. Herbs such as *L. barbarum* and *P. ginseng* should be administered with caution in damp-heat constitutions, while *G. jasminoides* and *L. japonica* require careful consideration in Yang-deficient individuals. Furthermore, a daily intake not exceeding 30 g of these food-medicine homologous herbs is generally recommended to ensure safety and minimize potential adverse effects.

The safety of herbal medicines has always been an important issue for scholars at home and abroad. In the United States, a significant portion of the population uses herbal medicines, and De Smet PA et al. (255) in 2002 showed that attention should be paid to the quality and safety of herbal medicines, and clinicians should also be concerned about the clinical efficacy and potential hazards of herbal medicines. Some literature reports that certain herbs are toxic. Toxicity reports often stem from long-term overconsumption, interactions with inappropriate foods or medications, or substandard quality (256, 257). For example, chronic or excessive ingestion of *G. alalensis* is discouraged, as evidence suggests a possible link to fluid retention and edema. Consequently, recognizing the broad range of medicinally applicable foods, we emphasize the importance of empowering DKD patients with knowledge and encouraging consultation with expert nutritionists and TCM practitioners to formulate individualized dietary strategies based on their unique constitution.

Although considerable potential exists, the current body of research investigating food-medicine homologous substances for the prevention and management of DKD is constrained by several key limitations. Firstly, there is a notable lack of innovation in the formulation of these herbal medicines, with studies being overly focused on isolated constituents while neglecting the holistic effects of the whole-food matrix. Secondly, clinical evidence predominantly stems from small-scale, short-duration studies that often lack well-defined outcome endpoints. Thirdly, the translation of basic research findings into clinical practice faces multiple challenges, including inadequate bioavailability of active ingredients, difficulties in standardizing herbal preparations, and considerable interindividual variability in treatment responses.

A paradigm shift and breakthroughs in translational research are urgently needed in the future. The following strategic directions are proposed: (1) Transition from a “constituent-driven” approach to a “whole-food/complex-system” research framework to systematically elucidate the synergistic mechanisms underlying their multi-target effects. This should be coupled with advanced formulation technologies, such as nano- and micro-encapsulation, to enhance bioavailability. Furthermore, rational combination strategies integrating food-medicine homologous herbs with conventional DKD therapies (e.g., SGLT2 inhibitors and RAAS blockers) should be explored. With a firm emphasis on safety, research should prioritize evaluating whether such combinations can yield synergistic renoprotective effects, mitigate adverse effects of conventional drugs, or improve overall metabolic management. (2) Future clinical trials should not only be large-scale and endpoint-oriented but also incorporate adaptive designs and account for diverse ethnic, genetic, and comorbidity backgrounds. Incorporating pharmacogenomic analyses and biomarker assessments within these trials will be critical for elucidating the determinants of interindividual variability and for informing the development of tailored treatment protocols. (3) The strategic convergence of multi-omics technologies, systems biology, and artificial intelligence (AI) holds significant promise. AI-powered drug repositioning, combined with integrated multi-omics profiling—spanning genomics, metabolomics, and gut microbiome analysis—can accelerate the identification of novel bioactive molecules from food-medicine sources, clarify complex polypharmacological mechanisms, and uncover biomarkers suitable for patient stratification. These methodologies will further support the creation of predictive models to direct individualized intervention strategies and bolster ongoing pharmacovigilance and long-term safety monitoring.

Through collaborative innovation, we aim to advance research on food-medicine homologous herbs, facilitating their transition from empirical use toward evidence-based, precise, and clinically translatable applications.

## 5 Conclusions

This review highlights 29 food-medicine homologous herbs with proven safety and efficacy in DKD. These herbs alleviate immune-inflammatory responses by modulating NF-κB, interleukins, TNF-α, chemokines, and adhesion molecules. They also reduce mitochondrial and non-mitochondrial ROS production, improving oxidative stress via Keap1/Nrf2/ARE, AMPK/SIRT, and NF-κB pathways. Renal fibrosis is suppressed through targeting fibrosis markers and regulating TGF-β/Smad and Notch signaling. Additionally, these herbs inhibit the AGEs/RAGE axis, correct gut dysbiosis, reduce apoptosis, activate autophagy, inhibit ferroptosis, and modulate microRNAs, collectively exerting renoprotective effects in DKD. Collectively, these findings underscore the therapeutic potential of food-medicine homologous herbs as integrative interventions for DKD and support their incorporation into evidence-based nutritional strategies.

## Glossary

ACR: albumin creatinine ratio
AGE: advanced glycosylation end-products
AI: artificial intelligence
AKT: protein kinase
AMPK: AMP-activated protein kinase
ARE: antioxidant response elements
ATF4: recombinant activating transcription factor 4
BAK: Bcl-2 homologous antagonist BAK
Bax: Bcl2-associated X protein
Bcl-2: B-cell lymphoma-2
CAT: catalase
CCR2: chemokine (C-C motif) receptor 2
CCL3: Chemokine (C-C motif) ligand 3
CdCl2: cadmium chloride
CKD: chronic kidney disease
Coll I: collagen type I
CTGF: connective tissue growth factor
DKD: diabetic kidney disease
ECM: extracellular matrix
EMT: epithelial mesenchymal transition
eNOS: endothelial nitric oxide synthase
ERK: extracellular regulated protein kinases
ETF: electron transfer flavoprotein
FN: fibronectin
FRU: fructose
FOXO: forkhead box O
GPx: glutathione peroxidases
GSH-Px: glucagon-like peptide peroxidase
GST: glutathione s-transferase
HFD: high-fat diet
HG: high glucose
HIF-1α: hypoxia inducible factor-1α
HK2: human kidney-2
HMCs: human mesangial cells
HO-1: heme oxygenase 1
ICAM-1: intercellular adhesion molecule-1
IDH2: isocitrate dehydrogenase 2 (NADP+)
IHC: immune histochemical, IKK, inhibitor of kappa B kinase
IκB: inhibitor of NF-κB
IL: interleukin
IL-1RA: interleukin-1 receptor antagonist
iNOS: inducible nitric oxide synthase
JNK: c-Jun N-terminal kinase
KDIGO: Kidney Disease: Improving Global Outcomes
Keap1: kelch-like ECH-associated protein 1
LKB1: liver kinase B1
MAO: monoaminoxidase
MAPK: mitogen-activated protein kinase
MCP-1: monocyte chemoattractant protein-1
MMPs: matrix metalloproteinases
Mn-SOD: Manganese superoxide dismutase
miRNAs: microRNAs
MPC-5: mouse podocyte cell-5
mRNA: messenger RNA
mTOR: mammalian target of rapamycin
NF-κB: nuclear factor kappa-B
NGAL: neutrophil gelatinase-associated lipocalin
NIK: NF-κB inducible kinase
NO: nitric oxide
NOS: nitric oxide synthase
NOX: nicotinamide adenine nucleotide phosphate oxidase
NQO1: NADPH: quinone oxidoreductase-1
Nrf2: nuclear factor erythroid 2-related factor-2
nNOS: neuronal nitric oxide synthase
8-OHdG: 8-hydroxydeoxyguanosine
PERK-eIF2α: protein kinase R-like ER kinase-eukaryotic translation initiation factor 2 alpha
PGC-1α: peroxisome proliferator-activated receptor-gamma coactivator-1alpha
PI3K: phosphatidylinositol 3-kinase
PKCb2: protein kinase C beta 2
RAGE: receptor for advanced glycation end products
ROS: reactive oxygen species
SCFAs: short-chain fatty acids
Sir: silent information regulator
SOD: superoxide dismutase
SphK1-S1P: sphingosine kinase 1-sphingosine 1-phosphate
STAT: signal transducer and activator of transcription
STZ: streptozotocin
αSMA: α-smooth muscle actin
TCM: traditional Chinese medicine
TECs: tubular epithelial cells
TFR1: transferrin receptor 1
TGF-β1/Smad: transforming growth factor beta 1/Smad
TIMPs: tissue inhibitor of metalloproteinases
TLR: Toll-like receptors
TNF-α: tumor necrosis factor-alpha
TNFR: tumor necrosis factor receptor
T-SOD: total superoxide dismutase
VCAM-1: vascular cell adhesion molecule-1
XO: xanthine oxidase.
